# Digital Image Analysis of Yeast Single Cells Growing in Two Different Oxygen Concentrations to Analyze the Population Growth and to Assist Individual-Based Modeling

**DOI:** 10.3389/fmicb.2017.02628

**Published:** 2018-01-04

**Authors:** Marta Ginovart, Rosa Carbó, Mónica Blanco, Xavier Portell

**Affiliations:** ^1^Department of Mathematics, Universitat Politècnica de Catalunya, Barcelona, Spain; ^2^Department of Agri-Food Engineering and Biotechnology, Universitat Politècnica de Catalunya, Barcelona, Spain; ^3^Cranfield Soil and Agrifood Institute, Cranfield University, Bedfordshire, United Kingdom

**Keywords:** *Saccharomyces cerevisiae*, morphometry, image analysis, aerobic, microaerophilic, Buchanan model, individual-based model, INDISIM-*Saccha*

## Abstract

Nowadays control of the growth of *Saccharomyces* to obtain biomass or cellular wall components is crucial for specific industrial applications. The general aim of this contribution is to deal with experimental data obtained from yeast cells and from yeast cultures to attempt the integration of the two levels of information, individual and population, to progress in the control of yeast biotechnological processes by means of the overall analysis of this set of experimental data, and to assist in the improvement of an individual-based model, namely, INDISIM-*Saccha*. Populations of *S. cerevisiae* growing in liquid batch culture, in aerobic and microaerophilic conditions, were studied. A set of digital images was taken during the population growth, and a protocol for the treatment and analyses of the images obtained was established. The piecewise linear model of Buchanan was adjusted to the temporal evolutions of the yeast populations to determine the kinetic parameters and changes of growth phases. In parallel, for all the yeast cells analyzed, values of direct morphological parameters, such as area, perimeter, major diameter, minor diameter, and derived ones, such as circularity and elongation, were obtained. Graphical and numerical methods from descriptive statistics were applied to these data to characterize the growth phases and the budding state of the yeast cells in both experimental conditions, and inferential statistical methods were used to compare the diverse groups of data achieved. Oxidative metabolism of yeast in a medium with oxygen available and low initial sugar concentration can be taken into account in order to obtain a greater number of cells or larger cells. Morphological parameters were analyzed statistically to identify which were the most useful for the discrimination of the different states, according to budding and/or growth phase, in aerobic and microaerophilic conditions. The use of the experimental data for subsequent modeling work was then discussed and compared to simulation results generated with INDISIM-*Saccha*, which allowed us to advance in the development of this yeast model, and illustrated the utility of data at different levels of observation and the needs and logic behind the development of a microbial individual-based model.

## Introduction

*Saccharomyces cerevisiae*, known as brewer's yeast or bread yeast, is one of the yeasts with the greatest economic and social impact. *Saccharomyces cerevisiae* is a facultative anaerobic yeast and a Crabtree-positive yeast. In the presence of oxygen and low glucose concentration (e.g., below 10 g/L) it usually uses oxidative metabolism, but ferments in higher glucose concentrations (e.g., above 10 g/L) regardless of oxygen concentration. Alcoholic fermentation is the most widely used in several industrial processes. When the conditions of the environment vary *S. cerevisiae* must adapt to the environmental changes being forced to pass in a short period of time from aerobic conditions to microaerophilic and anaerobic conditions at the end, changing the type of metabolism depending on the concentration of oxygen present in its neighborhood.

There is an increasing interest in yeasts because of the potentiality of whole cells. For some biotechnological applications, it is very important to obtain large amounts of yeast biomass (rather than ethanol, as happens in other types of applications). In order to obtain greater numbers of cells or larger cells with more cellular components usable in diverse industries, *Saccharomyces* must grow in a medium with oxygen available and low initial sugar concentration, to avoid the Crabtree effect. The yeast obtained is utilized as starter in fermented beverage industries, or as probiotic yeast with health benefit, and it is also used to obtain cellular components such as proteins and polysaccharides (e.g., glucans), which are of great value as functional ingredients in the food industry (Arevalo-Villena et al., 2017).

Like all microorganisms, *S. cerevisiae* has defined growth phases that characterize the temporal evolution of population size in a batch culture: adaptation or latency phase (lag phase), exponential or logarithmic phase (log phase), stationary phase, and death phase. The determination of the different growth phases of a culture can assist in the understanding of the changes experienced by microbial population and single microorganisms. Studies about yeast life-history traits involved in the adaptation to different environments are indispensable. Carrying capacity (maximum size of the population that can be supported upon the available resources), reproduction rate or intrinsic growth rate, and cell size are three life-history traits affected by the medium. For instance, understanding the causes of the variability and correlations of life-history traits in a yeast batch culture requires the analysis of the rate of resource uptake, which depends both on the amount of resources in the environment and on the activity of enzymes involved in the uptake (Spor et al., 2008); in that work, these three life-history traits were strongly affected by the glucose content in the culture medium, with obvious trade-offs between carrying capacity and growth rate, and between growth rate and cell size.

Morphometry, a branch of morphology that refers to quantitative analysis of form (size and shape), can be applied to unicellular microorganisms. In the case of *S. cerevisiae*, spheroidal cells, ellipsoidal cells, and sometimes cylindrical cells can be observed. The components of size and shape are obtained from a set of quantitative variables such as length, width, height, angles, etc. that can be analyzed statistically in order to summarize the changes undergone in the object of study, that is, the microbial cell (Bookstein, 1997). The morphometric analysis consists of three fundamental stages: image processing, acquisition of variables, and statistical analysis (Toro et al., 2010). It should be noted that the growth rate, mutation, and environmental conditions affect the size and shape of the yeast. For instance, when *S. cerevisae* grows in anaerobic conditions, cells are generally smaller than cells grown under aerobic conditions (Liesche et al., 2015). In addition, the morphology of the cells is closely related to their physiological state and their status in the cell cycle (Coelho et al., 2004). The relevance of cell size measurements to study the response of yeast cells submitted to various stresses has also been shown (Tibayrenc et al., 2010; Portell et al., 2011).

Modeling, from its broadest definition, is a very necessary tool to represent, analyze and discuss issues related to biological systems. The classical mathematical modeling based on continuous functions, derivable functions, differential equations, optimization methods, function adjustments, together with statistical modeling are by far the most widely used methodologies. Computational models are an interesting alternative to these methodologies and they are a modeling approach that is gaining pace to investigate microbial systems. Among them, the agent-based models or individual-based models (IBMs) are becoming more frequently used (Gorochowski, 2016; Hellweger et al., 2016; Jayathilake et al., 2017). In order to investigate a microbial system the above mentioned tools or methodologies are necessary, and can complement one another, providing additional information that benefits the overall modeling task. The diverse sets of experimental data, from macroscopy or population-level and from microscopic or individual-level, proceeding from the system itself, enhance the different modeling methodologies, since they provide the opportunity to deal with different types of observations of the same system.

Microbial IBMs are computational models that explicitly simulate autonomous living entities. Traditionally, they have not been deemed necessary to deal with microbial liquid cultures, usually assumed to be performed by axenic populations under perfectly homogeneous media; however, even clonal populations show biological heterogeneity in the individual behavior (González-Cabaleiro et al., 2017). Microbes (individuals) are treated as unique and discrete entities which have at least two independent properties plus their position in the medium. Rules are applied to define the individuals and the behavior of the medium; hence, the descriptor rule-based approach fits the methodology. The behavior of the population, of all existing individuals at any given time, emerges from the cumulative behavior of biotic interactions (among individuals) and abiotic interactions (between individuals and surrounding medium), which are interactions at individual-level. At the same time, the system-level dynamics constrain the behavior of the individuals. IBMs facilitate the understanding and formulation of the connection between individual microbes and properties at population level (e.g. heterogeneity, diversity, structure), as well as the interactions of microbes within the population and with their changing environment. INDISIM-YEAST is a microbial IBM that simulates a generic budding yeast, and it was used to assess the methodology for the investigation of this microbial population (Ginovart et al., 2007, 2011a,b). After that, a quantitative IBM which was focused on the fermentative (anaerobic) growth of the yeast *S. cerevisiae* was designed and termed INDISIM-*Saccha* (Portell et al., 2014). However, the fact that this model incorporated a fermentative (anaerobic) yeast metabolism limited its applicability for the study of some interesting biotechnological processes. Thus INDISIM-*Saccha* was extended and adapted to take into account the aerobic growth of *S. cerevisiae*, obtaining only some preliminary results at a population level, but not those results corresponding to an individual level of observation (Portell, 2014).

Microbial IBMs in general (Hellweger et al., 2016), and in particular INDISIM-*Saccha* (Portell et al., 2014) construct a virtual representation of a real system, which allows the characterization of the cells by means of their size, shape, and biomass. Thus, this type of model is capable of dealing with ideas that configure certain purposes of biotechnological applications using these individuals (their biomass and/or cellular wall components), facilitating the investigation of the system by modeling individual actions or behaviors directly linked with metabolic pathways, reproduction and viability, limited or stimulated by the local environmental conditions where the microbe is located. This representation of a microbial system allows the inclusion of diverse life-history traits involved in the adaptation of yeast to its environment, for instance, at population level, the reproduction rate estimated by the intrinsic growth rate or the carrying capacity (maximum size of population supported by the available resources), and, at the same time, at individual level, the cell size related to lifespan. Aging in the mother cell by means of an asymmetry and replicative lifespan and aging in the population by means of nutrient availability and chronological lifespan of the individual yeast cells (Cipollina et al., 2007; Carmona-Gutierrez and Büttner, 2014) are taken into account in the INDISIM-*Saccha* model. These different forms of yeast aging enable the control of population dynamics.

The need to connect experimentalists and modelers in general, and in particular, the combination of microbial IBMs and experimentation has recently been advocated as microbial individual-based ecology (Kreft et al., 2013; Hellweger et al., 2016). Microbial IBMs use data provided by individual-based observations but the integration of these data into the formulation and implementation of these models is not a direct task. A gap between modelers and experimentalists does really exist and efforts to bring together and encourage cooperation between both communities is indispensable (Hellweger, 2017; Succurro et al., 2017). Providing clear evidence of the utility of the experimental data for, and the needs and logic behind the IBMs can be a valuable and straightforward way of filling the stated gap.

The aims of this work are: (i) to obtain and analyze the results of the different morphometric parameters from the digital image analysis of yeast cells growing in two initial oxygen conditions (aerobic and microaerophilic cultures); (ii) to analyze the kinetic parameters of the population growth in order to detect the transition among lag, log, and stationary phases; (iii) to identify and connect the individual yeast states and population growth phases using individual-based and population-based experimental observations; (iv) to detect in both concentrations of oxygen the population growth phases with either larger cells or greater number of cells; (v) to explore and critically assess the data analysis developed in the improvement of the parameterization and calibration of INDISIM-*Saccha*, carrying out the testing of some model predictions, both at a population level and single-cell level.

## Materials and methods

### Experimental data

The experimental study was carried out using the *Saccharomyces cerevisiae* (LALVIN DV10, LALLEMAND Australia), a yeast strain with remarkable biotechnological characteristics. The medium used in the aerobic growth tests was composed of 10 g/L glucose, 3 g/L yeast extract, and 3 g/L of casein peptone. In microaerophilic conditions, the medium was supplemented with 0.5 g/L sodium thioglycolate and 0.001 g/L resazurin which acted by lowering the redox potential of the medium and redox indicator respectively. The media pH was adjusted to 3.5 with ortophosphoric acid. In aerobic conditions the inocula were cultured in 250 mL cotton-plugged flasks with 100 mL of the same media used in the experimental cultures. In microaerophilic conditions the inocula were cultured in 50 mL tubes completely filled with the described media. The inocula were incubated at 27°C and stirred magnetically for 72 h. The cultures for the experimental data were cultivated in a 1,000 mL flask, with 600 mL of the same fresh medium, inoculated with 0.1 mL of the preculture and also incubated at 27°C using a magnetic stirrer (300 r.p.m.) for approximately 30 h.

The experiments were performed with five replicates. Every 90 min a sample was extracted from four flasks to be analyzed, reserving the fifth flask for measuring the dissolved oxygen. Viable population, dissolved oxygen and glucose concentration were determined regularly throughout the 30 h of the study. Ethanol concentration was determined at 18 h and at the end of the study. Colony forming units were determined by using the pour plate method. Glucose concentration was measured by high-performance liquid chromatography equipped with RI detector (HPLC; Bekman). Ethanol concentration was determined with a Hewlett Packard 5890 Series II GC equipped with flame ionization detection using nitrogen as carrier gas. Initial dissolved oxygen concentration was determined with an oxygen electrode (OxyGuard, Handy Polaris).

### Acquisition of digital images

**Figure 1:**
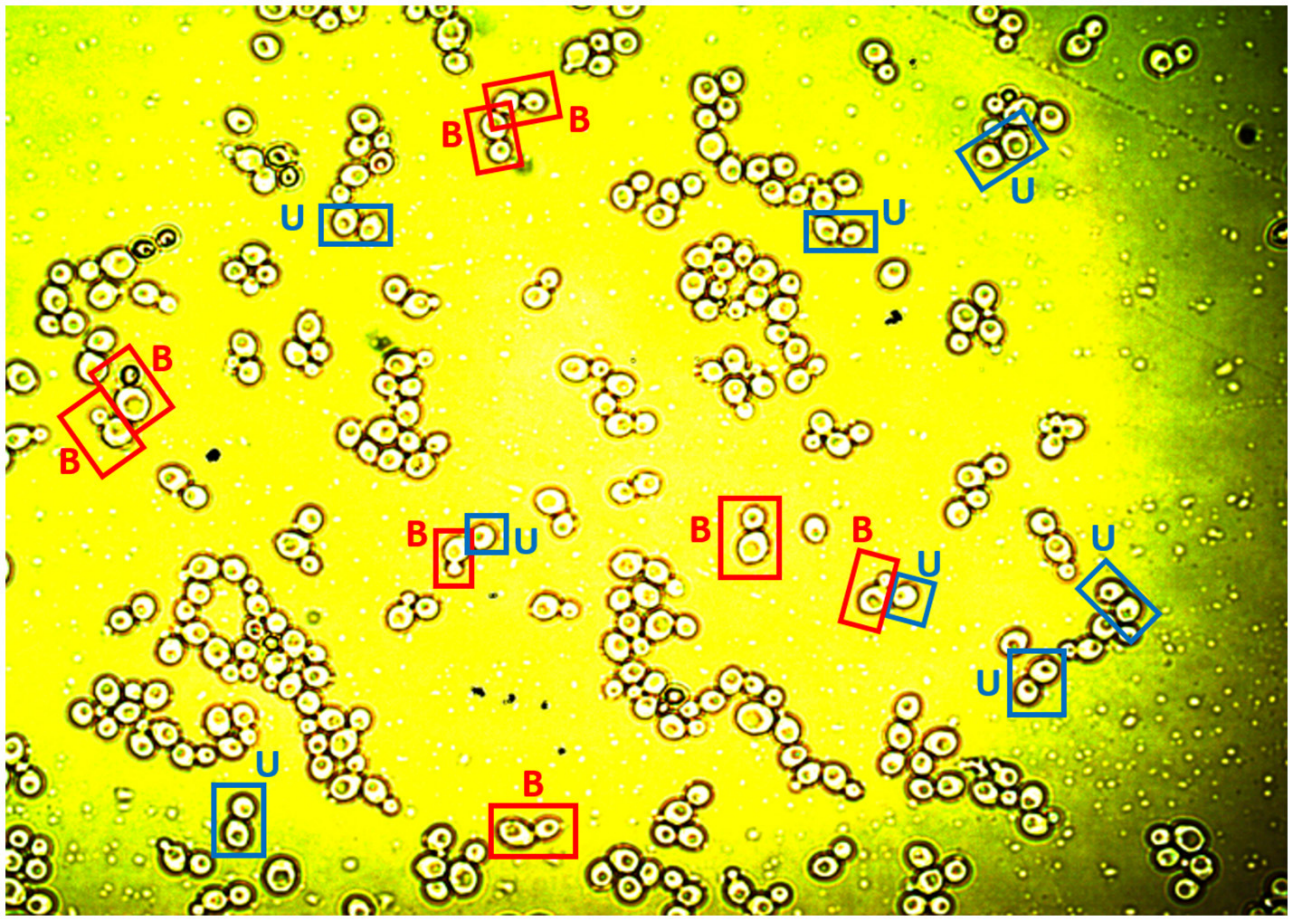
An image showing yeast cells available for analysis. The labels “B” and “U” exemplify cells classified as budded cells (B) or unbudded cells (U) after the analysis of the image (see text).

Images were taken using a Nikon Eclipse LV100 microscope equipped with a digital camera Nikon Infinity 1 Tv lens C-0.45x mounting an objective Nikon Lu Plan Fuer 50x/0.08° (numerical aperture). After calibration, the software Perfect Image v7.7 was used to obtain the images. The pixel size found was 0.0975 μm. Cell preparations were obtained every 90 min for the four replicates of the aerobic and microaerophilic conditions, producing three images per preparation. Samples were not sonicated before being processed (see also Supplementary Material). When necessary, the cell suspension was diluted in sterile saline solution and homogenized using a Vortex mixer. Figure 1 shows an example of the images obtained.

### Digital image analysis

**Figure 2:**
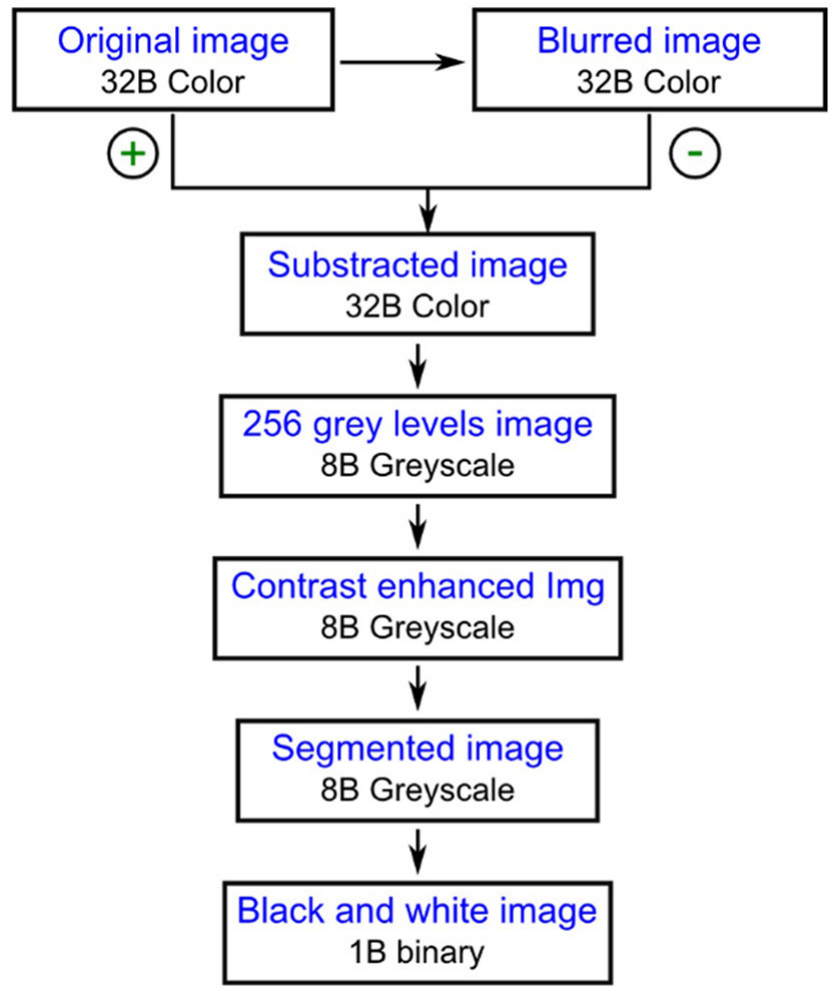
The first steps performed for the digital image analysis.

ImageJ is an open source image processing program for multidimensional image data with a focus on scientific imaging in the public domain (http://rsb.info.nih.gov/ij/index.html). Fiji is a distribution of ImageJ that focuses on biological-image analysis (http://fiji.sc/Fiji). To analyze all digital images obtained a protocol was designed and performed using the open source image processing package Fiji (Schindelin et al., 2012). The 32 B color images were transformed to binary files following the steps summarized in Figure 2. A blurred copy of the image was subtracted from the original image in order to decrease the noise coming from the background and the resulting 32 B color image was saved as an 8 B greyscale image. The image contrast was enhanced using the options “Saturated pixels” and “Normalize” from the “Enhance Contrast” tool. After segmentation using the option “Auto Threshold” from the “Adjust” menu of Fiji, the image was saved as a 1B black and white image. Four more steps were then applied to the binary image to obtain separated yeast cells: automatic object closing, hole filling, object separation, and manual object closing (Pertusa, 2010).

Each of the cells studied was analyzed individually to verify that the ImageJ program had complied with the criteria previously established in the protocol of the digital image analysis to separate the objects correctly. In order to identify the budded cells in the image analysis, in the case of pairs of cells, it was considered that the buds were those cells which were smaller than the one to which they were adhered. However, when a couple of cells were of similar sizes they were digitally processed as separate cells. Thus, from direct visual inspection of the images collected, the budding state (yes or not) of each one of the cells was manually recorded (as would have been done with the help of a microscope). Figure 1 shows some examples of the classification applied to the yeast cells as budded cells and unbudded cells.

Imaged objects (yeast cells) were characterized by measuring direct and derived morphological parameters. Direct morphological parameters studied were: the area (A), the perimeter (P), the major diameter (D_MAX_), and the minor diameter (D_MIN_). The derived morphological parameters computed and studied were circularity and elongation (or aspect ratio). The circularity (C) is a measure of the agreement of the shape of the object to a perfect circle (C = 1) that takes into account the area and perimeter of a cell, and can be computed as follows:

(1) $$\begin{array}{r} C=4\;\pi \;\frac{A}{P^{2}} \end{array}$$

The aspect ratio (AR) is the quotient between the major diameter and minor diameter of the ellipse fitting inside the object surface:

(2) $$\begin{array}{r} AR=\frac{D_{MAX}}{D_{MIN}} \end{array}$$

and it can also be regarded as a common measure of elongation (E) because values moving away from 1 indicate an increasingly elongated shape.

A macro was implemented in the ImageJ program to carry out the analysis of all the images in the most automatic way possible (fix the scale, introduce fixed values of some parameters, improve image contrast, etc.), obtaining better defined cells with no dirty spots or background noise around them. After performing several tests to adjust the threshold, three macros were created with three different methods for the “Auto Threshold” option included in Fiji. The most frequently used method was “Default,” although depending on the image, the options “Intermodes” or “Yen” were applied since they better defined the cells, with clearly delimited borders and less noise. However, once a macro was executed, to evaluate whether a manual intervention was necessary in this process, it was verified that: (i) all the analyzed objects were considered as cells of interest, since the program did not distinguish a yeast cell from other objects (such as air bubbles or suspended particles); (ii) the budded cells remained attached; and (iii) the correct execution of the closures of the edges of the cells was made.

### Analysis of data at both the microbial population level and the individual level of yeast cells

In a microbial system there are macroscopic observations that inform about the temporal evolution of the population, and microscopic observations that provide information on the specific characteristics of the cells making up this population. In this study both type of observations were accomplished and the investigation of the system was carried out by combining and comparing results on kinetic parameters of adjusted continuous dynamic models at the population level (macroscopic observations), and on models related to the distributions of individual properties of the elements that configure this population (microscopic observations). Thus, for the analysis of the data two different methodologies were applied according to the typology of the observations. In the first stage of the process, the modeling of the growth of the microbial population with the estimated kinetic parameters that characterized the temporal evolution was used. In the second stage of the modeling process, the individual variables (area, perimeter, minor diameter, major diameter, circularity and elongation) of the cells forming these populations were studied by means of the evolutions of their distributions. At this stage, the information obtained in the first stage of the population analysis was taken into account.

Both numerical summaries (descriptive statistics) and graphical summaries (boxplots, histograms, and scatterplots) were performed to synthesize and provide information for the two growth conditions (aerobic and microaerophilic). Possible association between pairs of variables were tested using the Chi-square test of independence.

From the temporal evolutions of number of yeast cells, the fitting of the three phase linear model known as Buchanan model (Buchanan et al., 1997) was carried out. It is a simple but good enough model for the purposes of this work, since it allowed us to estimate the kinetic parameters involved in the definition of the three main phases that characterize a typical curve of microbial growth in a closed liquid culture: lag phase, log phase, and stationary phase. In the Buchanan model, the three phases are described as follows:

(3) $$P\left(t\right)=\{\begin{array}{c} \begin{array}{c} \;P_{0}\;for\;t\leq t_{lag}\\ \; \end{array}\\ P_{0}+u\left(t-t_{lag}\right)\;for\;t_{lag}\leq t\leq t_{\max}\\ \;\\ \;P_{\max}\;for\;t\geq t_{\max} \end{array}$$

where: *P*(*t*)is the base 10 logarithm of population density at time *t* (Log cfu/mL); *P*_0_ is the base 10 logarithm of the initial population density (Log cfu/mL); *P*_*max*_ is the base 10 logarithm of the maximum population density maintained by the environment or the carrying capacity of the system (Log cfu/mL); *t* is the elapsed time (h); *t*_*lag*_ is the time at which the adaptation phase ends (h); *t*_*max*_ is the time in which the maximum population density is reached (h); and μ is the specific growth rate of the culture (Log cfu/mL h^−1^). The model describes the evolution of the population providing a mathematical method for the adjustment of growth curves with a good approximation to the way in which microbiologists have traditionally estimated the kinetic parameters of growth. The piecewise linear model of Buchanan was fitted to the experimental data with the nlsMicrobio package (Baty and Delignette-Muller, 2014) of the statistical program R (R Core Team, 2013). Point estimations of all the kinetic parameters involved in the definition of this model were obtained, identifying and characterizing the three growth phases, lag, log, and stationary, for each of the eight temporal evolutions available (four replicates for the two growth conditions).

To assess the influence of the medium conditions on the growth kinetic parameters obtained, the Student's *t*-test was used to compare the two means (independent samples for aerobic vs. microaerophilic), assuming or not equal standard deviations depending on the result of the Bonett test (for the comparison of variances).

In order to analyze the morphologic data acquired from the individual yeast cells, graphical and numerical summaries of the direct morphologic variables (area, perimeter, minor diameter and major diameter) were obtained, as well as of the variables derived from them (circularity and elongation). Data were grouped first by sampling times and growth conditions (aerobic or microaerophilic). Furthermore, the fact that each yeast cell belonged to one of the three different phases identified with the Buchanan model (for each replicate) contributed to the interpretation and comparative analysis of the results.

Contingency tables were used to study the independence between cell classification variables by considering the growth medium, the growth phase and the state of the cellular cycle (budded cell or unbudded cell). Scatterplots and linear correlation coefficients aided in the examination of linear and nonlinear relationships between the different variables. The analysis of variance (ANOVA) was applied to compare the means of the variables studied for the different groups determined by growth conditions, growth phase and bud state, followed by the corresponding separation of means. Nevertheless, because these data had unequal variances between groups the Welch's ANOVA test was run and subsequently Games-Howell method was used to compare all pairs of groups.

Discriminant analysis, a multivariate technique to classify observations into two or more groups from a data set with known groups (the training set), was applied to the data set of the four replicates taken together. It also helped to investigate how variables (predictors) contributed to group separation and to place individuals into defined groups (response). This can be used to develop rules for classifying other data sets for which group membership is not known. Linear discriminant analysis, one of the most commonly used techniques, assumes multivariate normality of the variables measured within each group and equal variances and covariances within each group. Using this model, linear discriminant analysis creates variables (discriminant functions) that are combinations of the original variables, which discriminate maximally between groups, and a quadratic analysis is used instead when the assumption of equal variances and covariances for all groups is not adequate (Sparks et al., 1999).

The program Minitab® 17 (2010) and the significance level 5% were used in the statistical analyses.

### INDISIM-*Saccha*: an individual-based model of the yeast *Saccharomyces cerevisiae*

The original INDISIM-*Saccha* model focused on the fermentative growth of *S. cerevisiae* and was introduced to the scientific community by the work of Portell et al. (2014), which was accompanied with Supplementary Material online, with a detailed description of INDISIM-*Saccha* and some significant aspects of the process used for the parameterization of this model. Here an overview of the modeling methodology used in the present work is shown. INDISIM-*Saccha* was developed to analyze the dynamics of *S. cerevisiae* in anaerobic batch cultures evolving in a non-stirred liquid medium with glucose as a main carbon source and organic and inorganic nitrogen sources. Global simulation scheduling consisted of initialization of the simulated system with the entrance of the input data, establishment of the initial configuration of the population, initial setting of the space, and the time step loop (which is repeated until the end of the defined time steps) including the random order of the individuals' acting order, the individual actions loop, the actions over the medium, and the output of variables. At each time step and at the individual actions loop, the existing yeast cells carry out, sequentially, the following set of actions: (i) random motion, (ii) uptake of the three substrates, namely, glucose, organic nitrogen and ammonium (controlled by the internal carbon to nitrogen ratio of the yeast cell) using size-based uptake submodel, (iii) metabolism with maintenance requirements, creation of carbon reserves, new mass synthesis, and release of substances, (iv) reproduction of mother cells and daughter cells, with a budding phase and an unbudded phase, and (v) lifespan (both chronological and replicative lifespan are considered).

INDISIM-*Saccha* assumes that the cellular cycle involves two differentiated phases. Phase 1, or unbudded phase, covers most of Gap1 phase (G1) and a very small fraction of synthesis phase (S) in the traditional division of the cell cycle; while Phase 2, or budding phase, covers a small fraction of G1, most of S and all of Gap2 phase (G2) and mitosis phase (M) (see Prats et al., 2010; Ginovart et al., 2011a,b, and references therein). Conceptually, the model assumes that in the unbudded phase the yeast cell is getting ready for budding and that change to the budding phase takes place only when the cell: (i) has attained a minimum cellular mass, defined by the parameter m_C_, the critical or minimum mass; and (ii) has achieved a minimum growth of its mass, which is related to the model parameter Δm_B1_. Within the model, two conditions must be satisfied for the releasing of the bud, and the subsequent change to the unbudded phase. These are: (i) a minimum growth of mass, which is related to the parameter Δm_B2_; and (ii) a minimum time interval, which is related to the parameter Δ_t2_.

For further comprehension of the model the reader is referred to the published work Portell et al. (2014).

#### Adaptation of INDISIM-*Saccha* to tackle aerobic conditions

The model was extended to deal with the analysis of dynamics of *S. cerevisiae* batch cultures evolving into a stirred aerobic liquid medium with glucose as a main C source, organic and inorganic N sources. This adaptation required the implementation of the following new features: (i) introduction of oxygen as a metabolic substrate for the yeast; (ii) utilization of aerobic or anaerobic catabolic pathways according to the local level of oxygen; and (iii) control of an individual lag time for the adaptation of the inoculum to new environmental conditions. When possible, model parameter values were taken following the work of Portell et al. (2014), but the new parameters had to be parameterized anew to guarantee a reasonably good reproduction of some experimental macroscopic results of the culture such as glucose, ethanol and cell density at the first stage of the iterative modeling process (Portell, 2014).

In INDISIM-*Saccha* a yeast cell is defined by a set of variables: the three Cartesians coordinates identifying its position in the domain; *M*(*t*), its structural mass (CN_MIC_-pmol); *B*(*t*), its genealogical age (bud scars); *Ph*(*t*), the reproduction phase in the cellular cycle in which the cell is currently (unbudded or budding phase); *M*_*Start*_(*t*), its “Start mass” (CN_MIC_-pmol), the mass required to change from the unbudded to the budding phase; *M*_*inc*_(*t*), the increased mass (CN_MIC_-pmol) since the cell entered to the budding phase; *T*_*inc*_(*t*), time spent into the current reproduction phase (time steps); *R*_*GLU*_(*t*), the amount of C stored in the cell as reserve carbohydrates or in the model as a glucose polymers (glucose-pmol); *R*_CN_(*t*), the amount of organic N stored in the cell as a reserve (CN-pmol); $\;C_{GLU}^{in}\left(t\right)$, amount of non-metabolized glucose inside the cell (glucose-pmol); and, *D*(*t*) the mortality index to evaluate cell viability. The values of these variables of all individual cells are stored internally and if required, can be used to generate individual based and global-based simulated observations. The environment simulates a liquid medium enclosed in a cube whose faces do not allow neither the ingress nor the egress of either organic or inorganic elements, with the exception of molecular oxygen that can be, or not, inflow to the system to maintain aerobic conditions. Four substrates can be taken up by the yeast cells: glucose (GLU), organic N (CN), ammonium (NH4), and molecular oxygen (O_2_), and ethanol and CO_2_ can be produced. The reproduction submodel of INDISIM-*Saccha* assumes that for a budded cell, the mass of the bud (M_Inc_) and the mass of the mother cell (M-M_Inc_) to be spherical. Therefore, it is possible to compute the radius of the mother (R_m_) and the bud mass (R_b_).

The metabolism submodel of this extended INDISIM-*Saccha* version considers the respiratory catabolic pathway (glycolysis and Krebs cycle) as the first option in achieving metabolic energy. Nevertheless, it is assumed that the cell can also use the fermentative catabolic pathway (glycolysis and alcoholic fermentation) once the uptaken O_2_ is depleted (or locally found at a very low level). This enables to control the level of O_2_ to fix growth conditions. Such an assumption allows the model to be used in aerobic growth conditions with low glucose content media, i.e., growth conditions not promoting a noticeable Crabtree effect.

An extra effort needs to be done in order to improve the parameterization of this new version INDISIM-*Saccha* able to tackle yeast growth with oxygen in the medium. The parameterization and calibration of the model will benefit from the individual-level data obtained with the digital image analysis protocol developed in the present work. A new output module was created so that the INDISIM-*Saccha* model could mimic the experimental output obtained in the present contribution. The stated module stored the reproduction phase (budded cell or unbudded cell), that is, the values of R_m_, and R_b_ of all the existing yeast cells at the requested sampling times. It is worth noticing that displaying the outputs in this way it is possible to simulate most of the morphologic measures obtained experimentally in this contribution.

## Results

### Image analysis of the yeast cells

Table 1 displays the number of cells analyzed in each experimental replicate for each sampling time for the two growth conditions, aerobic and microaerophilic.

**Table 1:** Number of cells analyzed for the different sampling times and for the four replicates (R1, R2, R3, and R4) under aerobic (A) and microaerophilic (M) conditions.

**Time (h)**	**AR1**	**AR2**	**AR3**	**AR4**	**ATotal**	**Time (h)**	**MR1**	**MR2**	**MR3**	**MR4**	**MTotal**
4.50	40	26	32	35	133	1.50	18	13	10	15	56
6.00	32	31	36	24	123	3.00	11	9	16	0	36
7.50	47	80	45	107	279	4.50	16	11	15	12	54
9.00	67	82	56	59	264	6.00	15	15	13	21	64
12.00	39	34	31	51	155	7.50	22	13	21	16	72
13.50	43	49	35	40	167	9.00	17	17	23	21	78
15.00	35	77	69	12	193	10.50	15	17	24	18	74
16.50	58	41	38	77	214	11.75	19	23	18	25	85
18.00	62	142	138	80	422	12.25	9	9	7	14	39
19.50	207	121	150	202	680	13.50	16	15	16	14	61
21.00	152	127	106	185	570	15.00	17	25	40	24	106
22.50	269	197	154	163	783	16.00	17	30	28	33	108
24.00	254	156	69	171	650	16.50	43	32	26	26	127
25.50	162	172	132	204	670	19.50	29	48	42	35	154
27.00	128	276	245	127	776	21.00	60	31	35	36	162
35.00	104	73	96	215	488	22.50	27	33	38	35	133
36.50	102	78	181	97	458	24.25	52	48	24	33	157
47.75	183	161	183	142	669	25.75	64	80	23	20	187
	1,984	1,923	1,796	1,991	7,694	27.25	22	64	36	44	166
						28.75	7	17	43	68	135
						30.25	71	77	56	59	263
							567	627	554	569	2,317

Before performing the statistical analysis of the obtained results it was necessary to debug the data set and eliminate some very extreme outliers.

**Figure 3:**
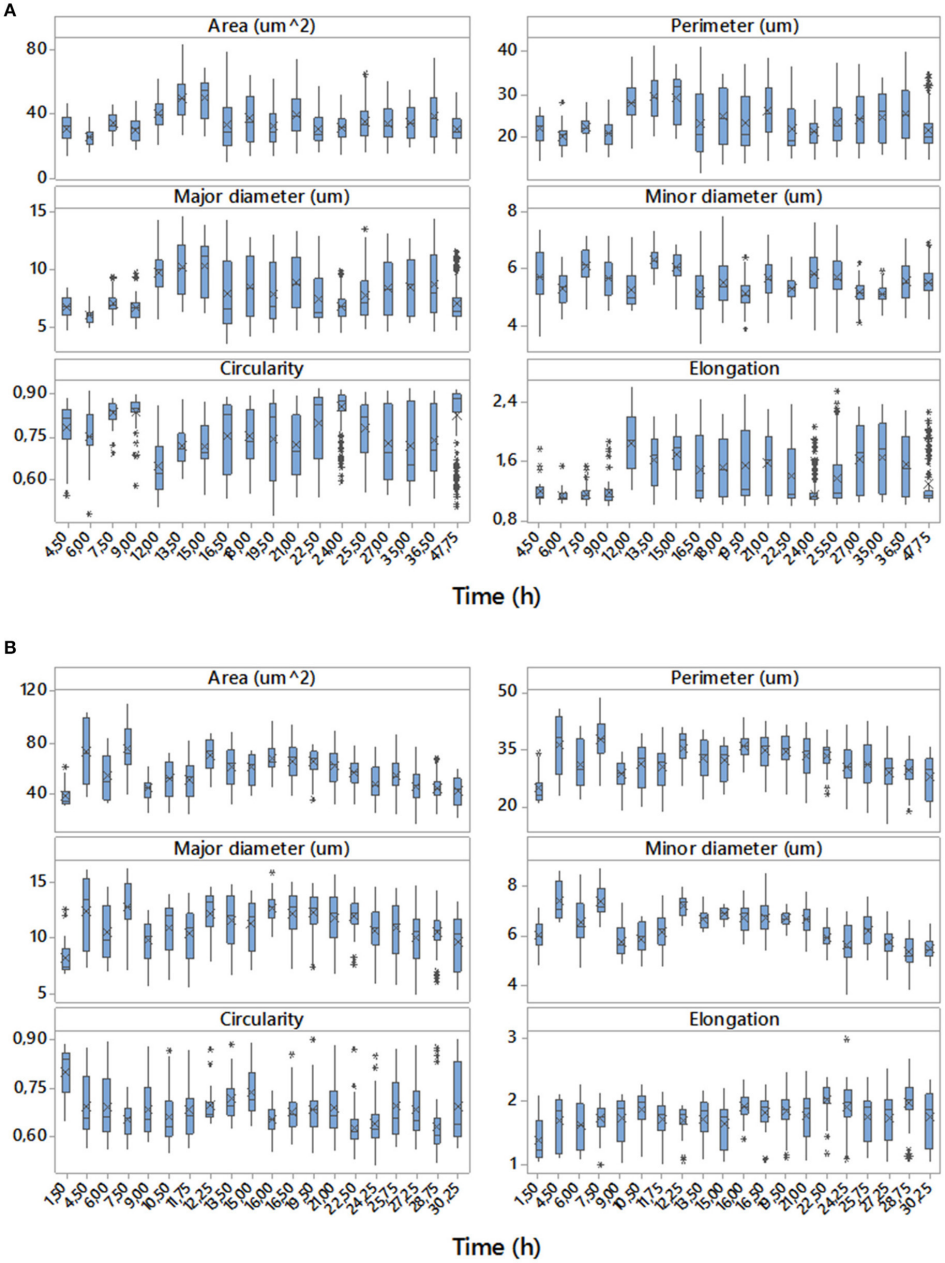
Temporal evolutions of boxplots for the data of the set of variables studied corresponding to replicate 1 with aerobic conditions **(A)** and replicate 4 with microaerophilic conditions **(B)**. On the boxplots, a line is drawn across the box at the median. Asterisks denote outliers (data that were more than 1.5 times the interquartile range above or below the box), and the “x” symbol represents the mean.

Examples of data achieved with the digital image analyses are shown in Figure 3. The sets of boxplots presented are useful for assessing and comparing sample distributions. They display the temporal evolutions of 50% of central data with the location of means and medians of the samples for each time corresponding to direct (area, perimeter, major and minor diameter) and derived (circularity and elongation) morphologic parameters studied for two of the eight replicates, one performed in aerobic conditions and the other in microaerophilic conditions. From the boxplots it is clear that the majority of the distributions are far from Gaussian distributions. These variables change their values, tendencies and variabilities along the temporal evolutions studied, and the values for the two growth conditions differ depending on the ranges used for those representations. These comments can be generalized to the rest of the experimental replicates with all variables (data not shown).

### Population kinetic parameters

**Figure 4:**
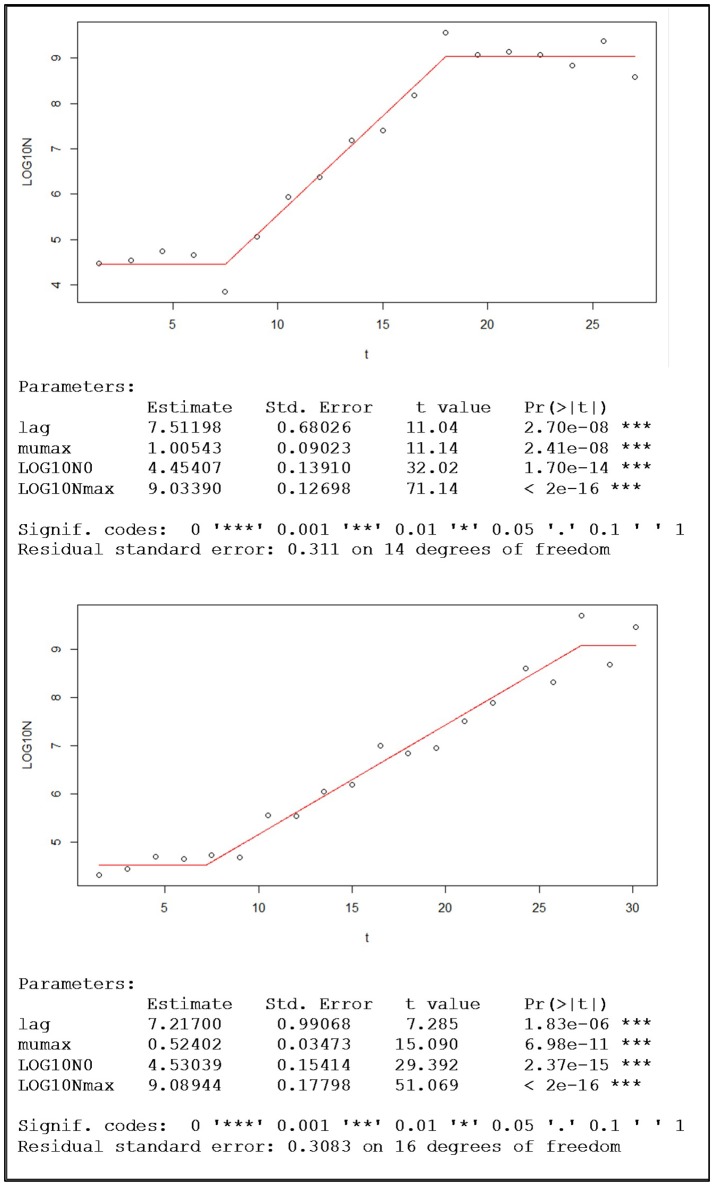
Examples of population growth, with the data of one replicate under aerobic conditions (top) and the other under microaerophilic conditions (bottom), fitted to the Buchanan's piecewise linear model and the sets of corresponding kinetic parameter estimations provided by the nlsMicrobio package of R, from the formula: LOG10N ~ LOG10N0 + (t ≥ lag) ^*^ (t ≤ (lag + (LOG10Nmax − LOG10N0) ^*^ log(10)/mumax)) ^*^ mumax ^*^ (t − lag)/log(10) + (t ≥ lag) ^*^ (t > (lag + (LOG10Nmax − LOG10N0) ^*^ log(10)/mumax)) ^*^ (LOG10Nmax − LOG10N0), where lag corresponds to time at which the change from lag phase to log phase occurs, the duration of the lag phase (h); mumax is the maximum rate of growth (Log cfu/mL h-1); Log10N0 is the logarithm of the initial population (Log cfu/mL); and Log10Nmax is the logarithm of the maximum population (Log cfu/mL).

Before fitting the Buchanan model the data were transformed using the base-10 (common) logarithm. The visual inspection of the adjusted piecewise linear model, together with the information generated by the nlsMicrobio package of the statistical program R, confirmed that for the eight replicates the quality of the fitting was very good. Figure 4 shows two examples of this type of adjusted model, one with aerobic conditions and the other with microaerophilic conditions, in which the estimates of the parameters involved are also displayed.

Once the parameters of the Buchanan model for each of the experimental replicates were estimated (Table 2), the transition time between the end of the log phase and the beginning of the stationary phase (t_Exp−Stat_) could be calculated by replacing the corresponding values of the estimates in the mathematical expression of the piecewise linear model. Furthermore, from this information the duration of the log phase for each replicate was estimated as the difference between the time of the entrance in the stationary phase and the duration of the adaptation or output of the lag phase. Taking into account the information in Table 2 each of the objects identified as a yeast cell with the digital analysis performed was labeled to indicate to which population growth phase they belonged. From the estimates of the kinetic parameters obtained by the fitting of the Buchanan model (Table 2), the temporal evolutions of the eight replicates were characterized.

**Table 2:** Summary of the kinetic parameters for the replicates of the aerobic conditions and the microaerophilic conditions with: t_Lag−Exp_, duration time of the lag phase or time at which the change from lag phase to log phase occurs (h); μ max, maximum growth rate (log cfu/mL h^−1^); Log10 (N0), logarithm of the initial population (log cfu/mL); Log10 (Nmax), logarithm of the maximum population (log cfu/mL), and t_Exp−Stat_, time in which the change from log phase to stationary phase occurs.

	**Aerobic conditions**					**Microaerophilic conditions**				
**Parameters**	**R1**	**R2**	**R3**	**R4**	**Mean ± StDev**	**R1**	**R2**	**R3**	**R4**	**Mean ± StDev**
t_Lag−Exp_	6.31	8.63	7.51	7.37	7.46 ± 0.95	6.79	7.95	7.22	7.14	7.28 ± 0.49
μ max	0.80	0.95	1.01	0.74	0.88 ± 0.13	0.54	0.60	0.52	0.55	0.55 ± 0.03
log10 (N0)	4.62	4.90	4.45	4.70	4.67 ± 0.19	4.48	4.60	4.53	4.59	4.55 ± 0.06
log10 (Nmax)	8.97	9.14	9.03	8.86	9.00 ± 0.12	9.16	9.19	9.09	9.57	9.25 ± 0.22
t_Exp−Stat_	18.83	18.89	18.00	20.34	19.02 ± 0.97	26.58	25.61	27.25	28.03	26.48 ± 0.82

In order to compare the kinetic parameters in the two growth conditions the Student's *t*-test for independent samples was applied. It can be concluded that the maximum growth rates for both conditions were significantly different (*p* = 0.016), with a greater growth rate in aerobic conditions. The means of the times in which the change from log phase to stationary phase took place were also significantly different (*p* < 0.001), with a greater time for microaerophilic conditions. Regarding the duration of the log phase, significant differences were also observed between the two growth conditions (*p* < 0.001), and, in particular, this duration was greater for the microaerophilic case. For the rest of parameters studied (initial population, final population and duration of the lag phase) it was concluded that the differences were non-significant at 5% (*p*-values were equal to 0.259, 0.747, and 0.086, respectively). Therefore, the evenness of mean values for these parameters in the two growth conditions was maintained.

### Descriptive analysis of the distributions of the morphologic parameters of yeast cells throughout the different phases of population growth

**Figure 5:**
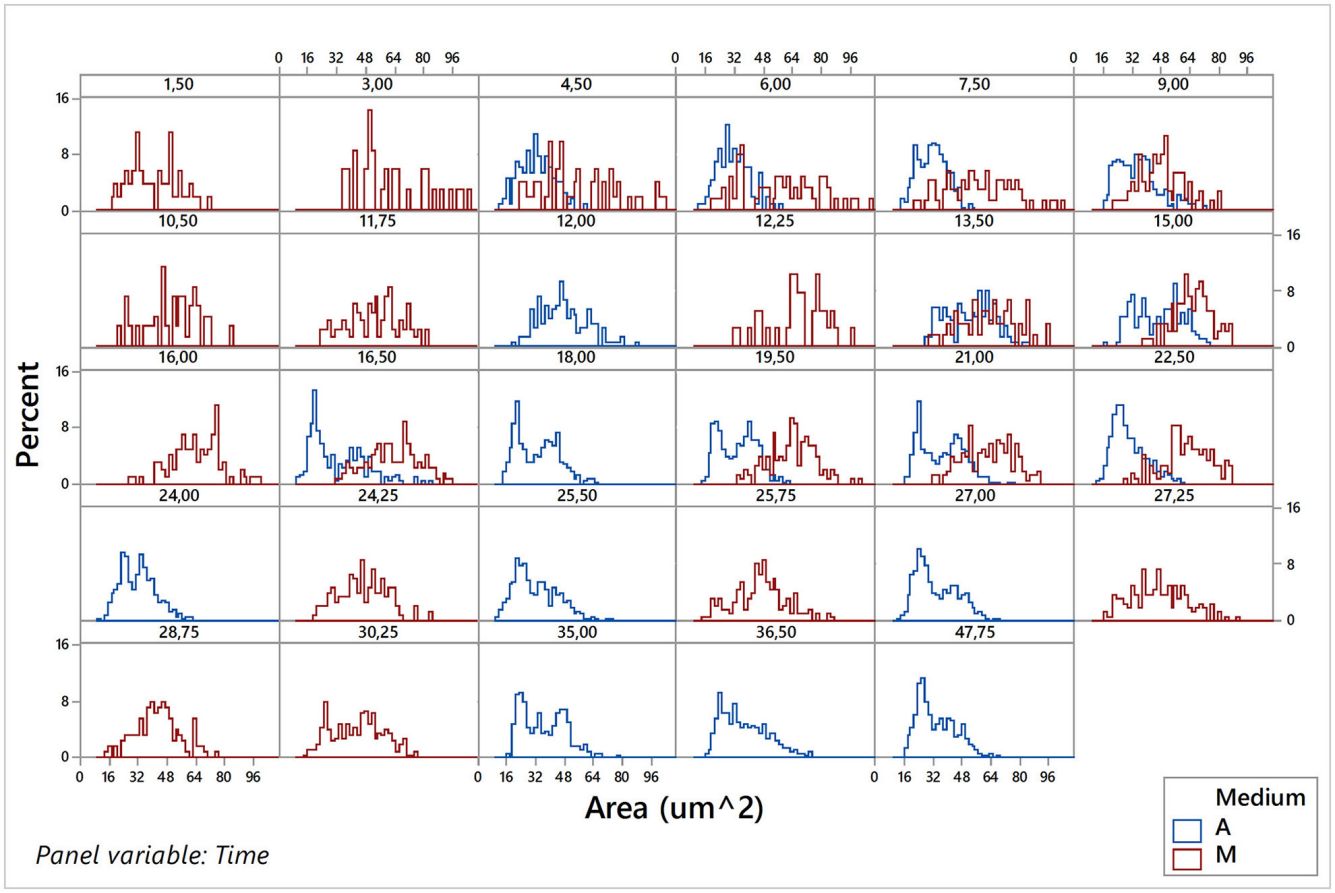
Histograms of the area variable for the pooled data of the four replicates under aerobic conditions (A) and of the four replicates under microaerophilic conditions (M) corresponding to different sampling times.

Considering that sampling of images was carried out during the population growth, and using the estimations of the kinetic parameters obtained previously with the Buchanan model, it was possible to locate each of the sampling times for each of the eight replicates (4 aerobic and 4 microaerophilic) to one specific population growth phase (lag, log, or stationary). Once the data sets debugged, the graphical representation and characterization of the distributions for the different morphologic variables according to the sampling times were conducted for each of the replicates of the two growth conditions tested, were conducted (data not shown). Since no noteworthy differences were detected between the behaviors observed for the four replicates in each of the two growth conditions, the data of the four replicates for each time sampling for both conditions were combined and analyzed for both conditions. Hence, changes in the central trends of distributions according to the growth phase, changes in variability or range of distributions, as well as changes in the form of distributions, were much better appreciated and more evident. Figure 5 and Figures S2.1–S2.4 of the Supplementary Material illustrate this type of information obtained from the individual analysis of the cells.

Figure 5 shows the variable area of cells. In aerobic conditions the areas range from 20 to 70 μm^2^ and that the distribution of the percentages vary according to the phase of growth in which the population is. Mainly in the lag phase (sampling times at 4.5, 6, and 7.5 h), but also in the stationary phase (sample times greater than 20 h), there is a higher percentage of cells of areas smaller than 45 μm^2^. However, during the log phase there is an increase in the percentage of cells with a larger area (greater than 45 μm^2^), some of them even reaching up to 75 μm^2^. In general, the areas followed approximately normal (Gaussian) distributions, with the exception of the end of log phase and beginning of the stationary phase that presented bimodal distributions. In these distributions, the smaller areas showed the highest frequencies. In microaerophilic conditions most of the areas range of values from 20 to 80 μm^2^ and even, in some cases, reach values of 95 μm^2^ in the first samples, which correspond to the lag phase, yet with lower percentages. Shape and central tendency of the distributions of the areas change slightly according to the growth phase. During the lag phase (sampling times less than 9 h) and stationary phase (samples corresponding to times beyond 27 h), the highest percentages correspond to values of low and medium areas (<55 μm^2^). In the log phase, the distribution of the cell areas shifts toward intermediate and high values (60–80 μm^2^). The distributions of areas in the lag and log phases are, for the most part, rectangular distributions, but at the end of the log phase and in the stationary phase the distribution tends to be about normal. The values of the areas may be associated with the growth phase, and the differences are better appreciated in aerobic rather than in microaerophilic conditions. In general, it is also observed that the cells in the lag phase and stationary phase are smaller than those in the log phase. The temporal distributions of the cell perimeters (Figure S2.1 in Supplementary Material) display in general similar behaviors to those observed with the cell areas (Figure 5). Figure S2.2 in Supplementary Material presents histograms of the major diameter and minor diameter of the cells. The temporal evolutions of the variables estimating shape, namely, elongation (or aspect ratio) and circularity, are displayed, respectively, in Figures S2.3, S2.4, which can be found in the Supplementary Material.

### Relationships between morphologic variables of yeast cells and phases of population growth according to the state in the reproduction cycle

It is evident that the studied variables of shape and size are considerably affected by the state of the cellular reproduction cycle. Taking into account the direct visual inspection of the images collected and the manual data recorded on the budding state of each of the cells, the dichotomous variable budding (Yes/No) was incorporated to the analysis.

Chi-square test for independence was used to assess the relationship between the two categorical variables (budding, growth phase) from the contingency table or cross tabulation of the pooled data (the two growth conditions together). As was expected, there was a strong evidence of association between the two variables (*p* < 0.001). For the 582 cells belonging to the lag phase only 184 were budded cells (31.6 %) and for the 5881 cells belonging to the stationary phase only 2719 were in the budding phase (46.2%), while in the log phase from the 3144 cells controlled, 2221 cells were in the budding phase (70.6%).

In order to analyze the association of these two variables, and to characterize their distributions, the raw data were split according to the budding phase, and the subsequent distributions were studied again. This separation of the data affected the shape, location and variability of the morphologic distributions in a very remarkable way. After having introduced the variable budding in the analysis, the distributions became much more regular and symmetric, approaching Gaussian distributions. The analysis of variance (ANOVA) was used to compare the means between the different subgroups obtained from the combination of these three factors (growth conditions, growth phase and budding reproduction). As the assumption of equal variances between groups was violated, Welch's one-way ANOVA test was performed. Once it was concluded that the means of subgroups were significantly different for all the variables (all *p*-values < 0.001), then the Games-Howell test was used to compare differences between all pairs of groups. Table 3 provides a numerical summary of the variables according to these three factors studied.

**Table 3:** Number of yeast cells (N), means (± standard deviations) of the groups formed with the combinations of the three factors (growth condition, population growth phase, individual state in the reproductive cycle) with the grouping mean information using the Games-Howell Method (95% confidence).

**Conditions-Phase-Budding**	***N***	**Area (μm)**	**Perimeter (μm)**	**Minor diameter (μm)**	**Major diameter (μm)**	**Elongation**	**Circularity**
A-lag-N	303	29.65 ± 7.98 g	21.46 ± 3.21 f g	5.70 ± 0.84 e	6.50 ± 0.91 g	1.15 ± 0.09 d	0.80 ± 0.07 d
A-lag-Y	61	39.24 ± 9.05 d	26.04 ± 3.61 d	5.79 ± 0.77 d e	8.58 ± 1.40 d	1.50 ± 0.27 c	0.72 ± 0.06 e
A-log-N	760	26.44 ± 7.71 h	19.62 ± 3.06 h	5.40 ± 0.77 f	6.12 ± 1.00 h	1.14 ± 0.10 d	0.85 ± 0.04 a b
A-log-Y	953	46.34 ± 11.82 c	29.58 ± 4.01 c	5.59 ± 0.73 e	10.45 ± 1.71 c	1.88 ± 0.29 b	0.66 ± 0.07 f
A-stat-N	3,027	26.25 ± 6.54 h	19.48 ± 2.61 h	5.39 ± 0.67 f	6.12 ± 0.85 h	1.14 ± 0.10 a	0.86 ± 0.04 a
A-stat-Y	2,276	43.20 ± 9.12 d	29.25 ± 3.63 c	5.32 ± 0.59 f	10.28 ± 1.49 c	1.95 ± 0.28 d	0.63 ± 0.06 h
M-lag-N	95	38.93 ± 13.82 d e	24.20 ± 4.86 d e	6.35 ± 1.04 a b c	7.66 ± 1.78 e	1.21 ± 0.25 d	0.82 ± 0.07 c d
M-lag-Y	123	65.58 ± 19.10 a	35.27 ± 5.47 a	6.72 ± 0.97 a	12.24 ± 2.19 a	1.83 ± 0.28 b	0.65 ± 0.06 f g
M-log-N	163	33.93 ± 8.29 e f	22.41 ± 3.00 e f	6.12 ± 0.80 c d	6.96 ± 0.93 f	1.14 ± 0.10 d	0.84 ± 0.04 b c
M-log-Y	1,268	60.67 ± 13.93 a	34.05 ± 4.34 a	6.35 ± 0.76 a b	12.05 ± 0.74 a	1.91 ± 0.25 b	0.65 ± 0.06 f g
M-stat-N	135	30.69 ± 9.06 f g	21.16 ± 3.40 g	5.75 ± 0.88 e	6.66 ± 1.05 f g	1.16 ± 0.11 d	0.85 ± 0.04 a b c
M-stat-Y	443	48.90 ± 13.05 b	30.95 ± 4.60 b	5.59 ± 0.80 e	10.99 ± 1.81 b	1.98 ± 0.29 a	0.63 ± 0.06 g h

*Where: M, microaerophilic condition; A, aerobic condition; lag, adaptation phase; log, exponential phase; stat, stationary phase; Y, budded cells; N, unbudded cells*.

*Means that do not share a letter are significantly different*.

**Figure 6:**
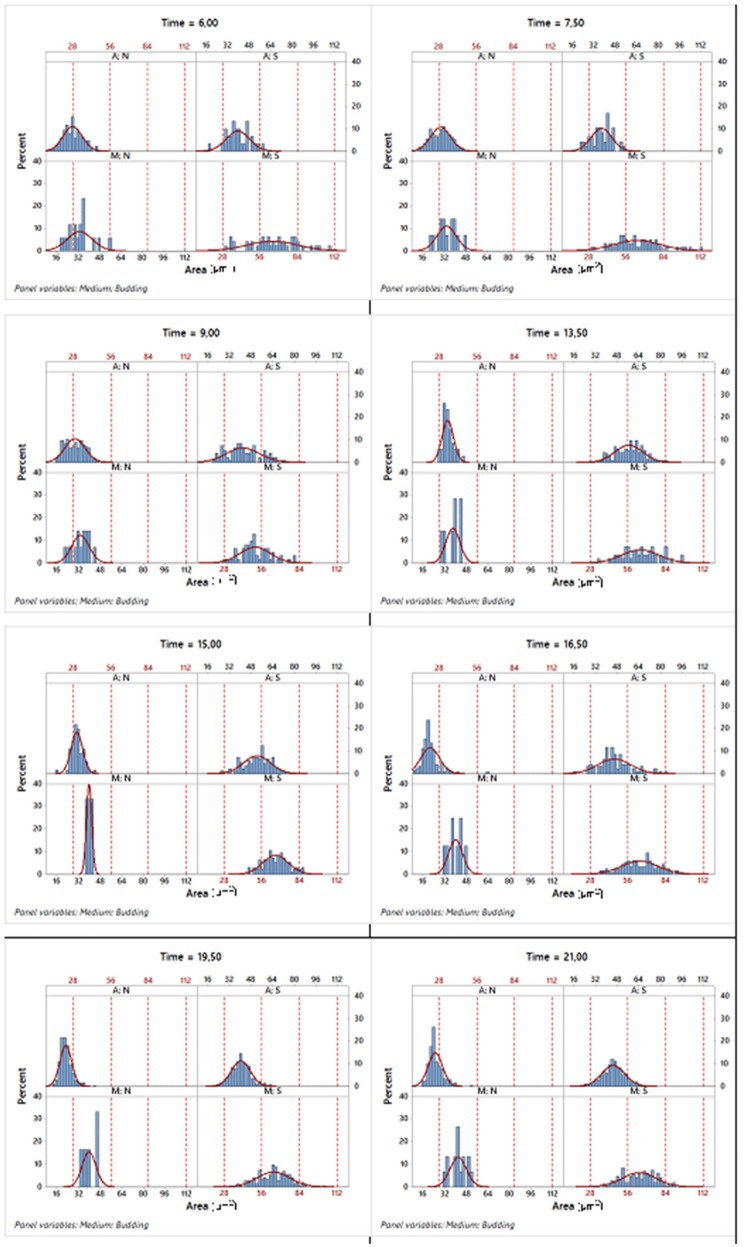
Histograms of the corresponding area variable in different sampling times where the data were split according to the two growth conditions and state in the reproduction process. A, aerobic conditions; M, microaerophilic conditions; S, budded cells and; N, unbudded cells.

For instance, regarding the area variable, Figure 6 displays a set of histograms corresponding to different sampling times, presenting the budded and unbudded cells separately for each of the two growth conditions.

Table 3 shows that for each phase, the mean of the areas of the unbudded cells in microaerophilic conditions were greater than those in aerobic conditions, and the same applies for the budded cells. In general, the means of the areas of budded cells were higher than those of unbudded cells, although not always significantly different. In relation to the means of the areas according to the growth phase, the budded cells in microaerophilic growth did not present significant differences between lag and log phases, nor when comparing the unbudded cells. Nevertheless, in aerobic conditions there were significant differences between the means of areas for lag and log phases in both cases (budded and unbudded cells). In the stationary phase for budded and unbudded cells and in both growth conditions, the means of the areas were reduced, although not always with significant differences. The highest values for the mean of the area were achieved by budded yeast growing in microaerophilic conditions during the lag and log phases with values of 65.6 and 60.7 μm^2^ (not significantly different). The means of the perimeters behaved similarly to the means of the areas. About the variability of the area distributions, the biggest coefficient of variation (35.5%) was for the unbudded cells grown in microaerophilic conditions that were in the lag phase (followed by those cells that were in the stationary phase, with a value of 29.5%), while the smallest coefficient of variation of the area distributions (21.1%) was for the budded cells grown in aerobic conditions that were in the stationary phase. The group of budded cells grown in microaerophilic conditions in the log phase had a small coefficient of variation (23%).

Regarding the three growth phases, the means of the minor diameters in aerobic conditions behaved differently from those in microaerophilic conditions (Table 3). The mean of the minor diameters of the unbudded cells under aerobic conditions in the lag phase was higher than those in the log and stationary phases. The latter two means showed no significant differences between them. The means of the lag and log phases behaved similarly in both growth conditions. Regarding the major diameters, the means followed the same pattern in the three growth phases. The means of unbudded cells in aerobic conditions showed the lowest values, while the highest values corresponded to the budded cells in microaerophilic conditions in lag and log phases. Unbudded cells in microaerophilic conditions, and budded cells in aerobic conditions showed significant differences according to growth phases. The means of the major diameters of the lag phase were higher than the means of the log and stationary phases, except in the case of budded cells under aerobic conditions. In microaerophilic conditions the means of budded cells in the lag and log phases were significantly different from that in the stationary phase.

From direct visual inspection, a set of 5124 budded cells was gathered, with a mean elongation value of 1.92 and a coefficient of variation of 14.6%. Considering the data of the two growth conditions together, the 95% confidence interval of the mean elongation was (1.91, 1.93). The median of this data set was 1.95, and the 50% of the central values for elongation of budded cells ranged between 1.76 and 2.12. However, the means of elongation corresponding to the different phases of growth were 1.72, 1.90, and 1.95 for the lag, log and stationary phases respectively, with their corresponding 95% confidence intervals (1.68, 1.77), (1.89, 1.91), and (1.94, 1.96) respectively. No significant differences were observed for unbudded cells, regardless of the growth phase or the growth conditions, obtaining the smallest values. Under microaerophilic conditions the elongation had always higher values than under aerobic conditions. Taking into account the budded cells, for the log and stationary phases no significant differences were detected between both growth conditions, while for the lag phase the elongation means were significantly different. The mean elongation of the lag phase and budded cells in aerobic growth was much lower than the rest of budded cells with a value of 1.5 (Table 3).

In both growth conditions, the means of the circularity values of the unbudded cells were higher than those of the budded cells, and significantly different, with values clearly far from 1 (Table 3). Considering the unbudded cells, the highest value observed was 0.86 and the smallest was 0.80 corresponding to cells in the stationary phase and in the lag phase for aerobic growth conditions. In microaerophilic growth and unbudded cells there were no significant differences of the means of circularity according to the growth phases. The means of the circularity of the budded cells were different depending on the growth phase in aerobic conditions. The differences for this variable in function of the phases were smaller in microaerophilic growth than in aerobic growth.

### Discriminant analysis in aerobic and microaerophilic conditions

All the variables can be considered to be approximately normally distributed within each group, except circularity and elongation. Therefore, these two variables were excluded from the analysis. Since equal variances could not be assumed, a quadratic discriminant function was used, and there was no need to jackknife or cross-validate the results because the data set was sufficiently large (Sparks et al., 1999).

First, the data set of the cells was used in an attempt to discriminate between the two growth conditions (aerobic, microaerophilic) on the basis of their morphometry (area, perimeter, major diameter, minor diameter). The overall percentages of the cells that could be correctly allocated to aerobic and microaerophilic conditions were: area (78.5%), perimeter (70.2%), major diameter (69.0%), and minor diameter (73.8%), with partial percentages of correct classification in each group ranging from 60.9 to 82.1%. If allocations to growth conditions were completely at random one would expect 50% correct allocation. When two predictors were combined, the percentages were slightly improved. In particular, it was worth considering the combinations: area-perimeter (80.4%) and major–minor diameters (76.5%), because the former is connected with circularity, and the latter with elongation. The four-predictor combination was disregarded due to collinearity. Since the morphometric predictors showed a strong potential to discriminate between growth conditions, discriminant analysis to classify cells according to phase (lag, log, stationary) and to budding (Yes–No) is discussed below for each growth condition separately. The results are displayed in Table 4. It should be remembered that if allocation to groups budding and phase were completely at random, one would expect a 50% and a 33.3% correct allocation, respectively. From Table 4 it is clear that in both growth conditions, all the predictors, except minor diameter, showed a high potential to classify cells into groups according to their budding condition. The main differences in the discriminant power were detected when allocating cells to their growth phase. In microaerophilic conditions all predictors presented overall percentages much larger than 33.3%. However, the partial percentage of cells in lag phase correctly classified fell far below 33.3%, except for predictors minor diameter, and the combination major–minor diameters, with all the partial percentages in each phase above 33.3%. On the contrary, in aerobic conditions the overall percentages did not achieve 33.3%, except for the minor diameter. Nevertheless, even in this case not all groups were well classified. A more detailed analysis revealed that cells in stationary phase were not correctly classified, in general. To achieve a better discrimination in aerobic conditions, phase and budding were merged into a new group phase-budding, with six categories. Hence, if cells were allocated at random, one would expect a 16.67% correct allocation. The overall percentages improved in general: area (46.3%), perimeter (48.7%), major diameter (48.0%), minor diameter (24.9%), area-perimeter (53.4%), major-minor diameters (54.9%). It is worth pointing out that only for major diameter and area-perimeter all the groups showed partial percentages above 16.67%.

**Table 4:** Quadratic discriminant analysis of morphometric predictors, showing allocation of cells to groups (phase, budding), in aerobic and microaerophilic conditions.

		**Group**	
**Percentages correct (overall)**	**Predictors**	**Phase**	**Budding**
Aerobic	Area	18.4	85.0
	Perimeter	18.2	92.0
	Major diameter	18.2	94.7
	Minor diameter	57.9	50.4
	Area–Perimeter	33.1	95.3
	Major diameter–Minor diameter	28.0	97.0
Microaerophilic	Area	56.0	79.6
	Perimeter	59.5	89.1
	Major diameter	57.2	91.3
	Minor diameter	52.4	62.8
	Area–perimeter	52.8	94.1
	Major diameter–Minor diameter	57.0	94.4

### Simulations with the individual-based model INDISIM-*Saccha*

The IBM INDISIM-*Saccha* was extended by the incorporation of an oxygen-using metabolic alternative (see section Adaptation of INDISIM-*Saccha* to Tackle Aerobic Conditions). The preliminary simulation results aimed to test whether the adapted model was also capable of tackling experimental cultures with oxygen available, combining outputs at population level and at individual level in order to take full advantage of the experimental data recently achieved and previously analyzed.

**Figure 7:**
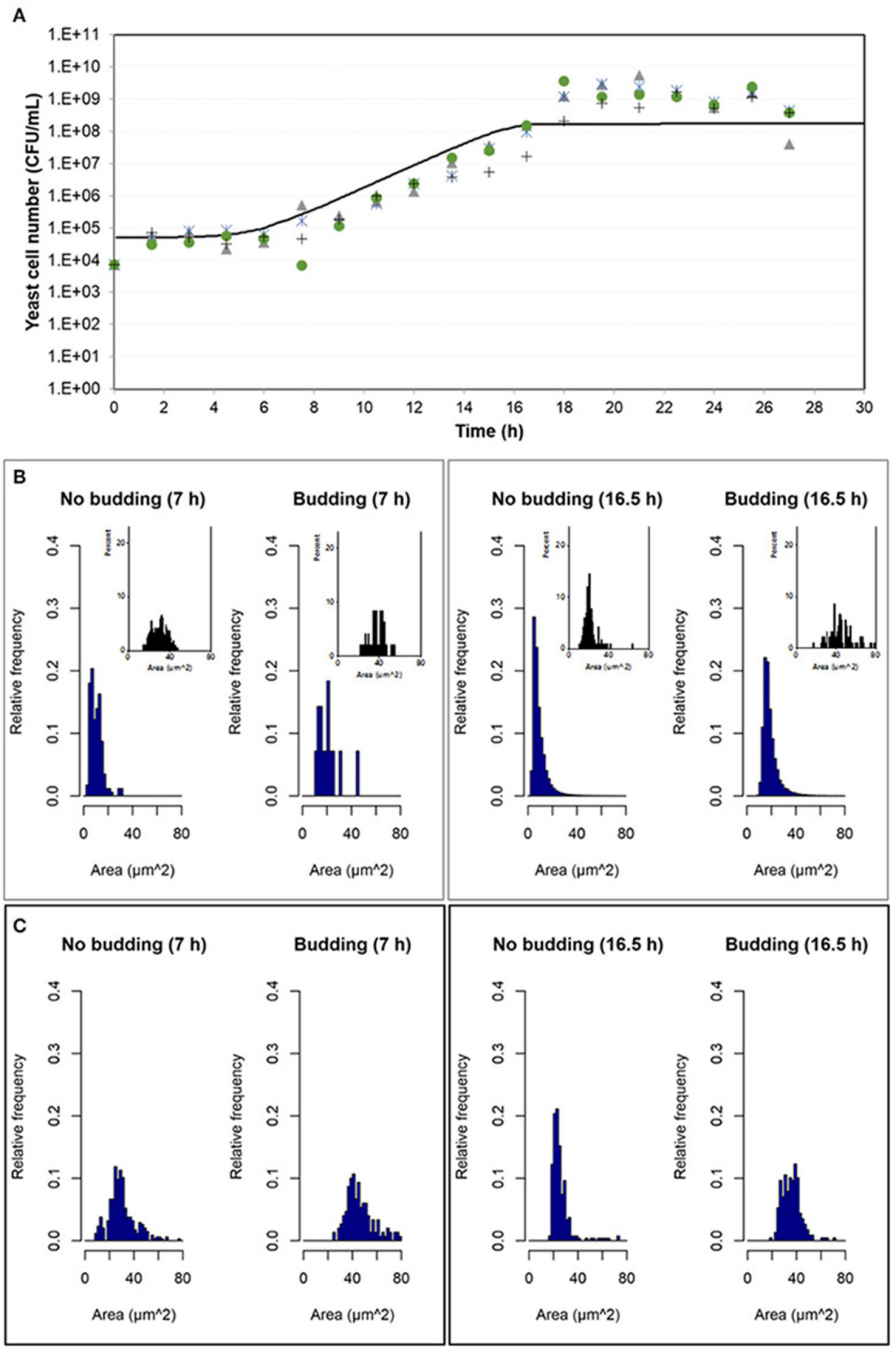
Outputs obtained in aerobic conditions. **(A)** Temporal evolution of the experimental data of the four replicates of the population growth (different symbols) with a simulation performed with INDISIM-*Saccha* (continuous line). **(B)** Distributions of the areas of the yeast cells that make up the virtual system (*in silico* population) at different sampling times with the data split according to the state in the reproduction process: S, budded cells and N, unbudded cells. Notice that the representation of the corresponding experimental results by means of small plots have been included in the same area of the simulated plots to facilitate the comparison. **(C)** Size distributions that were obtained by changing the value of the standard variability of the minimum reproduction mass (σ_mC_, from 0.15 to 0.25), the minimum growth required for the cell to start the budding process (Δm_B1_, from 0.5 to 2.0), and the minimum growth required for the bud to detach itself from the mother cell (Δm_B2_, from 1.0 to 9.0) and its standard deviation (σ_mB2_, from 0.25 to 0.02), while keeping the rest of the parameter values used in the simulation displayed in **(A,B)**. In addition, the size distributions in **(C)** were produced by randomly sampling 500 individuals from the simulated yeast population to better mimic the procedure used to produce the experimental distributions.

**Figure 8:**
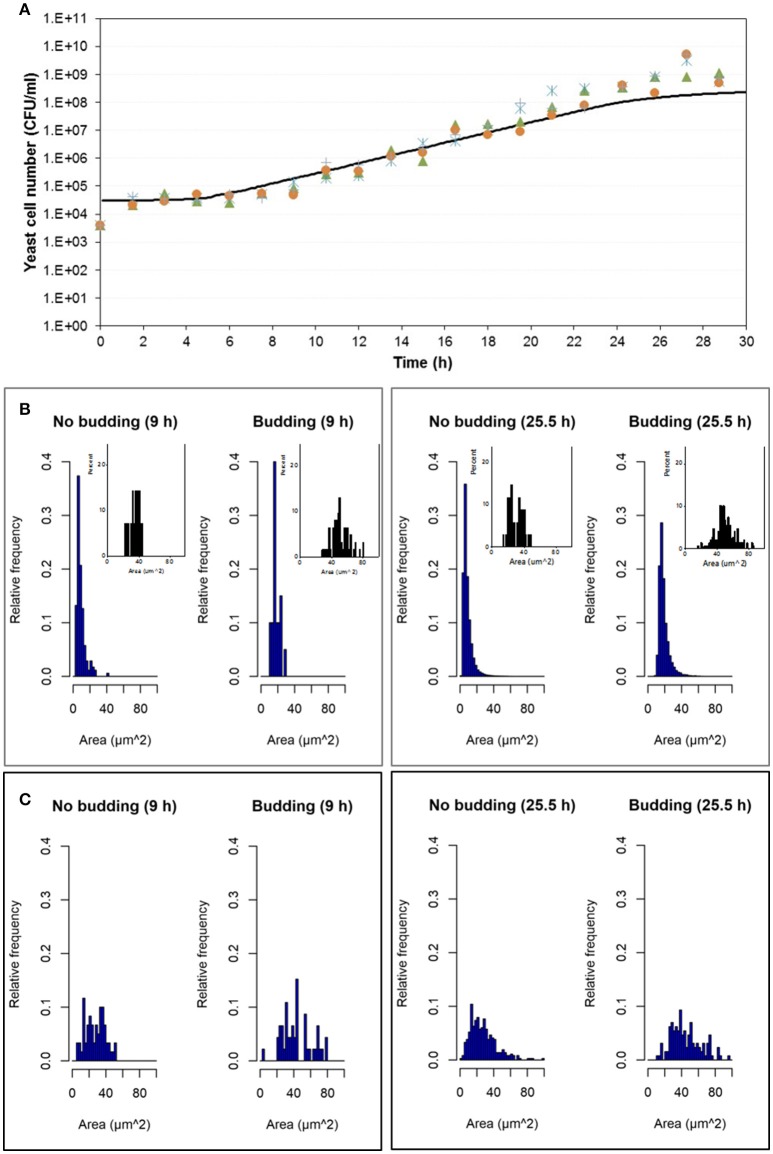
Outputs obtained in microaerophilic conditions. **(A)** Temporal evolution of the experimental data of the four replicates of the population growth (different symbols) with a simulation performed with INDISIM-*Saccha* (continuous line). **(B)** Distributions of the areas of the yeast cells that make up the virtual system (*in silico* population) at different sampling times with the data split according to the state in the reproduction process: S, budded cells and N, unbudded cells. Notice that the representation of the corresponding experimental results by means of small plots have been included in the same area of the simulated plots to facilitate the comparison. **(C)** Size distributions that were obtained by changing the value of the minimum reproduction mass (m_C_, from 5 to 15), of its standard variability (σ_mC_, from 0.15 to 0.5), the minimum growth required for the bud to detach itself from the mother cell (Δm_B2_, from 1.0 to 25.0) and of its standard deviation (σ_mB2_, from 0.25 to 0.75) while keeping the rest of the parameter values used in the simulation displayed in **(A,B)**. In addition, the size distributions in **(C)** were produced by randomly sampling 500 individuals from the simulated yeast population to better mimic the procedure used to produce the experimental distributions.

Experimental results were compared with simulation results at population level by means of the number of cells in the population growing in aerobic conditions and in microaerophilic conditions as Figures 7A, 8A respectively show. Nevertheless, with the study performed on the experimental distributions of the individual characteristics of yeast cells offered new options and challenges to be explored. A preliminary exploration of the outputs at individual level related with the mass of the yeast cells was carried out. Distributions of areas and volumes for budded and unbudded cells were recorded (see section INDISIM-*Saccha*: An Individual-Based Model of the Yeast *Saccharomyces cerevisiae*). A direct comparison can be drawn between simulation and experimental data. The simulated size distributions corresponding to the 7.0 and 16.5 h for aerobic conditions are shown in Figure 7B. Similarly, for microaerophilic conditions the simulated size distributions obtained at the sampling times 9.0 and 25.5 h are shown in Figure 8B. As can be seen, the area distributions from the simulated population followed unimodal distributions that change during time. Comparing the simulated distributions to their experimental equivalents a number of remarks can be made. First, simulated distributions during the lag phase (results not shown) did not change while changes were evident in the distributions of areas, perimeters, minor and major diameters, as well as in the derived morphologic variables (elongation and circularity) as Figure 5 and Figures S2.1–S2.2 display. Second, simulated both budded and unbudded cells were smaller than those observed experimentally, and the amplitude of the distributions found experimentally was greater; and, in addition, the simulated distributions were well formed (Figures 7B, 8B).

Several sets of simulations were carried out modifying the values of the parameters of the reproduction submodel. When these values increased, both mean cell sizes and amplitude of the simulated distributions of budded and unbudded cells increased and became closer to the experimental values. However, there was a fundamental difference between the experimental and the simulation sampling procedures that partially explained the discrepancies observed. The fact that a small allicot of the experimental procedure was being measured against the whole simulated population suggested that the sampling effect had to be accounted for also in the simulations. Consequently, samples of the virtual system were also generated in order to represent the simulated size distributions. Figures 7C, 8C show these improved simulation results. Nevertheless, focusing on the glucose, ethanol and cell number temporal evolutions, the agreement between experimental and simulated values became poorer (results not shown).

## Discussion

A collection of digital images of *S. cerevisiae* cells growing in two different initial concentrations of oxygen was processed to perform subsequently the statistical analysis of a set of morphologic parameters. A working protocol was established for the treatment of digital images of yeast cells using the free program ImageJ-Fiji, adjusting the parameters when necessary and designing different macros to automate the procedure. It turned out that the automation of the image analysis was not always the most suitable method, nor did it guarantee the thorough analysis of all the cells. Therefore, an individualized and manual review of all the analyzed cells was carried out, including the supervision of the corresponding morphologic parameters and the budding state with the visual inspection.

Concerning the type of model selected for the population growth, the Buchanan model proved to be very appropriate to fit the data analyzed, since in all the microbial cultures the different growth phases could be clearly identified (adaptation-lag, exponential-log, and stationary). The temporal experimental evolutions of yeast populations for the aerobic and microaerophilic conditions were well characterized from the estimations of the parameters provided by the fitting to the Buchanan model and from derived calculations. All the kinetic population parameters obtained from the model (durations of the lag and log phases, maximum growth rate, final population or carrying capacity, times of change from the log to the stationary phase) are of great microbiological interest for specific applications in biotechnology. However, it is necessary to point out that the values for the kinetic parameters also depend on the adjusted model (Buchanan et al., 1997; López et al., 2004; Griffiths et al., 2016). This fact should be taken into account when comparing experimental results with simulated results, as well as when referring to published results.

The production of starter cultures, a remarkable biotechnological application, using a batch process with a respiratory metabolism in microaerophilic conditions, according to the results obtained, allows the log phase to be extended, thus obtaining fully active and ready cells to be used as starters for a longer period of time. Therefore, the achievement of a long-term log phase of a population is positive for the industry. Starter populations that are in stationary phase suffer from a delay in their metabolic activity in the adaptation phase and in longer fermentative processes (Ekberg et al., 2013). In addition, in the stationary phase there is an aging of the population and a reduction of viable cells as a consequence of lack of nutrients. When the situation persists it causes the damage cell accumulating and cell death (Carmona-Gutierrez and Büttner, 2014).

Likewise, the population level parameters assisted to the interpretation of the morphologic parameter distributions of the yeast cells collected from the analysis of the digital images during the successive population growth phases. According to the purpose of the yeast cultivation, the study of growth conditions advantageous for the attainment of either a high number of yeast cells or larger cells is noteworthy. For both the kinetic parameters of the population growth and the distributions of the individual variables of size and shape of the cells, it was shown that there were important differences between the two growth conditions tested. This reinforces the idea that a microbial system should be analyzed from different perspectives (population - individual) in order to make the most of the available information in any modeling process. This two-fold analysis is indispensable and much more relevant in any iterative process of parameterization and calibration of IBMs. It has also been established that the individual information obtained experimentally should be coupled with that obtained at the population level for the same system since, for slightly different levels of oxygen significant differences in population parameters were detected, as well as in the distributions of individual variables of the yeast cells that made up those populations.

In both aerobic and microaerophilic growth conditions, the numerical summaries of central tendency and variability obtained for the area, perimeter, major and minor diameters, elongation and circularity of the yeast cells were studied together with their respective distributions, which were not always normally distributed. Analysis and comparison of the distributions of these morphometric variables allowed to connect them with the three main microbial growth phases (log, lag, and stationary). These distributions reflected the changes between population growth phases in both growth conditions, and in a more relevant way for the aerobic growth, probably due to a faster growth and greater differentiation between phases.

The distributions of the size variables such as area and perimeter presented similar evolutions. The results obtained showed that cells growing in microaerophilic conditions presented larger areas than those growing under aerobic conditions. There were wider ranges for the distributions of the budded cells than for those corresponding to the unbudded cells. In general, the greatest cellular sizes occurred in the log phase, in both oxygen conditions, in keeping with other studies (Powell et al., 2003; Dungrawala et al., 2012), although in certain conditions their values get closer to those achieved in the lag phase.

The values of the major diameters, and to a lesser extent, those of the minor diameters, reproduce again the evolution of the cell size in the different growth phases, as observed in the variables area and perimeter. As the log phase progressed, the percentage of cells with a larger diameter increased, although percentages of smaller cells (new cells) were also maintained. The bimodal distributions in the log phase of all size parameters (except for the minor diameter) indicated the presence of two groups of cells differentiated by sizes that could be related to mother cells and daughter cells, the latter not having yet reached critical size to bud. The increasing percentage of small cells detected in the stationary phase has been described in several studies (Aragon et al., 2008; Svenkrtova et al., 2016) and may be related to glucose depletion. But it may also be connected with the presence of cells that in the stationary phase could give rise to quiescent cells (Li et al., 2013; Carbó et al., 2015). Cells in microaerophilic growth did not present the two size-differentiated subpopulations at the end of the log phase and at the onset of the stationary phase, although a wide range of cell sizes could be observed, probably due to the slower and asynchronous growth. In general, they were larger than in aerobic growth at all phases. The small reduction of the initial oxygen concentration in the medium, such as the one proposed in this study, led to larger *Saccharomyces* cells. This could mean an improvement in the industrial production of cells for dietary supplements or cellular derivatives, such as glucans used in the bakery industry, or for their bioactive properties in the pharmaceutical products (Freimund et al., 2003). Under microaerophilic conditions larger cells were obtained and foreseeably with greater concentration of some cell components (although for some type of components this should be checked, in general it is true for cellular wall components such as glucans as they, β1-6 and β1-3 glucans, which constitute about 55–65% of the wall dry weight of the cell wall, Klis et al., 2002) It is worth bearing this result in mind if the purpose is to produce cells to extract cell metabolites. Besides, the populations grown in microaerophilic conditions were more homogeneous than those grown in aerobic conditions. On the other hand, a similar cellular concentration (biomass) was obtained in both growth conditions, but in aerobic conditions this concentration was achieved from 8 to 10 h before that in microaerophilic conditions, which indicates a higher yield in the aerobic case. Nevertheless, the consumption of glucose was superior in aerobic conditions, which makes it more expensive to obtain biomass in industry [the glucose in aerobic growth was exhausted at 18 h, just at the beginning of the stationary phase, whereas at the beginning of the stationary phase of the microaerophilic growth, there were still 6.68 g/L of glucose (data not shown)].

Differences in shape were also detected (see Figures S2.3, S2.4 of the Supplementary Material). Cells in microaerophilic growth presented mainly cylindrical or more elongated shape, whereas those in the aerobic conditions were mostly oval or elliptic. The elongation and circularity variables provided information on the deformation of the cells. Coelho et al. (2004) proposed an elongation value of 1.5 for *S. cerevisiae*. Although this reference value must be calculated for each microorganism growing under specific conditions, it held for the yeast cells in this study, in both growth conditions, hence allowing the discrimination between budded and unbudded cells. Differences in elongation values depending on the growth phase could be in agreement with the changes of the cell size for the different growth phases. Regarding the circularity, it was more difficult to establish a value that allowed to discriminate so clearly the budded cells from the unbudded cells, since apparently it depended on the growth phase. The discriminant analysis supported that, in both growth conditions, size assisted in classifying cells according to their budding state. However, while in microaerophilic conditions size could accurately allocate cells to their growth phase, in aerobic conditions only the combination of growth phase with budding state granted an adequate discrimination.

Both oxygen concentrations studied affected the growth rate, cell size and to a lesser degree, the number of viable cells of *Saccharomyces* obtained at the end of the study. There is a trade-off between the growth rate and the cellular size similar to that shown by Spor et al. (2008) when studying the influence of different concentrations of glucose. It was observed that with a higher concentration of oxygen dissolved in the medium, a higher growth rate was detected, while cell sizes were smaller; but, conversely, with lower initial oxygen concentration, a lower growth rate appeared while cell sizes were greater. There was no trade-off between the growth rate and the final viable population achieved unlike the results shown in the work of Spor et al. (2008); a higher initial oxygen concentration resulted in a greater growth rate and a greater number of cells achieved in less time (while more time was required to achieve the same final number of cells with a little less initial oxygen). Probably this behavior is due to the fact that the two initial oxygen concentrations considered in this study did not constitute a stress factor.

Key aspects that should be further developed to move microbial community modeling toward greater predictive power have recently been revised (e.g., Song et al., 2014). In many cases, in a microbial context, it is not yet understood how individual cells should be programmed, manipulated or cultivated to ensure the emergence of the required collective behavior. IBMs, together with a suitable experimental work, makes it easier to tackle these issues, offering a framework in which to simulate such systems.

The number of studies on IBMs addressing bacterial populations greatly exceeds those dealing with yeast populations (Hellweger and Bucci, 2009). However, there are, to our knowledge, a few microbial IMBs that have been used to tackle diverse questions related to yeast such as the work of Hellweger et al. (2014) who investigated the hypothesis of a fitness benefit of age-correlated stress resistance of yeast, or the work of Momeni et al. (2013) who examined how through partner fidelity feedback heterotypic cooperation between yeasts may be protected against cheaters. Studies performed with INDISIM-YEAST and INDISIM-*Saccha* focused on the qualitative behavior and on the patterns and tendencies of variables connected with the yeast system and their effect on the growth phases of the population, specifically on the duration of the lag phase (Ginovart et al., 2007, 2011a,b) and on the fermentative (anaerobic) growth (Portell et al., 2014) respectively. Based on individual actions and parameters for individual yeast cells rather than fitting the model to data, these IBMs could predict the measured compounts profiles as well as biomass and genealogical age distributions. With the current experimental information gathered and from the examination of the distributions of sizes and shapes of individual yeast throughout the different phases of population growth, a quantitative study was carried out in order to improve the parameterization and calibration of the new aerobic version of INDISIM-*Saccha*. The individual-level observation of size is an important parameter involved in the uptake submodel, which takes into account the available nutrient at a microscale patch and the probability of it encountering and entering the yeast through the cellular membrane. Likewise, the review of the budding reproduction submodel could be performed since it allowed not only to distinguish mother yeast cells from daughter yeast cells, but also to control the budding process, that is, the times and masses for the separations of the buds from the cells. Besides, the consideration of the cellular membrane of the individual yeast (which is related with its size and geometry) in the uptake submodel has effect on the amount of nutrients that the cell uses. The uptake submodel, which assumes that a yeast cell translocates low molecular weight compounds dissolved in water through its cell membrane, could be revisited in light of these findings about sizes and shapes of yeast cells. Such a revision would not be possible without the availability of hands-on experimentation as the one presented in this contribution.

There are several models that allow for mathematical descriptions of distributed cell properties within microbial populations, and cell size is usually chosen as a model variable to study yeast populations due to its tight coupling to cell growth and division, which in the case of this microorganism is asymmetric (e.g., Hatzis and Porro, 2006; Lencastre Fernandes et al., 2013). Nevertheless, an IBM grants the representation of biological actions for a microorganism and its integration into the structure of the model, and thereby cell size is indirectly involved in the individual behavior rules. In consequence, the available resources achieved by the virtual cell are shared between maintenance, creation of new biomass (size growth) and reproduction (increasing the size of the bud during the budding phase), which mostly determines the distribution of sizes that the model provides. Moreover, the distinction between timers and sizes, two classical concepts for G1 control, was investigated in yeast cells, and it turned out that size-independent noise (presumably molecular noise) is the leading source of variability in the duration of G1 (Di Talia et al., 2007). Thus, a deterministic size control model would seem insufficient, being then appropriate to incorporate certain stochasticity at individual cell level to achieve virtual representations of yeast populations when mass distributions and dynamics are explored. This reinforces the idea that stochastic IBMs, such as INDISIM-*Saccha*, are necessary tools to integrate both biological and environmental heterogeneity to improve the process design and scale up of microbial processes (González-Cabaleiro et al., 2017).

In the INDISIM-*Saccha* model, yeast cells experienced an individual lag time, that is, a period in which they were internally adapting in order to be able to undergo cellular growth. In the simulation, the size of the cells undergoing the lag phase did not change since the model assumed they were suffering the internal changes required to start growing. Such behavior was chosen for the sake of simplicity but there exist other conceptualizations that can be regarded as plausible in the literature (Prats et al., 2008, 2010). The behavior observed in the simulations does not seem to agree completely with the experimental findings. Although at the population level and during the adaptation phase no movement of the total number of cells was perceived, at individual level, as Figure 5 and Figures S2.1–S2.4 showed, changes were evident in the distributions of areas, perimeters, minor and major diameters, as well as in the derived morphologic variables (elongation and circularity). This seems to suggest that the submodel describing the lag time of the individual cells should be reviewed in such a way that would allow for more variability and changes in the temporal evolution of the distribution of the individual areas, and, eventually, validated against the tendencies observed in the present contribution.

The development of a calibration procedure incorporating cell size distribution at strategic time points during the different growth phases will help reach a better agreement between both kinds of data (experimental and simulated). It should be pointed out that the digital analysis procedure that we have developed lets sorting the size of budded and unbudded yeast. To our knowledge this approach has not been used so far, yet it offers some interesting characteristics that render it suitable for a calibration step. In particular, it allows to isolate more efficiently the effect of changes on the model parameters of the reproduction submodel so they can be detected more easily. For instance, in relation to the second simulation result (Figure 7C) from the first simulation result (Figure 7B) of the size distributions, fine tuning a model parameter, mainly controlling the size of the bud before detaching it from the mother cell (i.e., Δm_B2_), will affect exclusively the distribution of the budded cells, but not the distribution of the unbudded yeasts. Other parameters, such as the critical mass before starting the budding phase (Δm_B1_), will have an impact on the distribution of both budded and unbudded yeast cells.

The comparison between the individual-level information, obtained from the digital analysis procedure, and the simulation outputs of a calibrated IBM of the yeast *S. cerevisiae* is a valuable approach to test the accuracy of the process undergone. Although this does not invalidate the usefulness of the calibrated model for particular goals, the model obviously is not able to capture a number of important aspects of the real system. From now on, and with the current experimental information accomplished, a quantitative study can be carried out in order to improve the calibration of the new INDISIM-*Saccha* from the particular examination of distributions of sizes and shapes of individual yeast throughout the different phases of population growth.

This type of study on microorganisms is essential to ponder and develop methodologies for calibration processes of models to tackle different levels of observation of the system under consideration. Making IBMs simultaneously reproduce patterns observed at both the individual and population level will make these IBMs structurally realistic so that they can deliver independent, testable predictions (Kreft et al., 2013; Hellweger et al., 2016). The tasks to complete this experimental-modeling-experimental iterative process require a close relationship and extra effort to connect both experimentalists and modelers, this approach being exemplified by the model INDISIM-*Saccha*. Neither of the two levels of observation (population and individual) in the process of parameterization and calibration can be neglected, although this requires an extra effort for modelers and an increment in the complexity of the models (Hellweger, 2017).

The combination of individual-level knowledge, gathered from the digital images processed, with population-level information, drawn from primary growth models and the estimation of kinetic parameters, proves to be crucial in understanding the biological processes connecting different experimental observations. The design, parameterization, calibration and validation of a microbial IBMs can certainly benefit from this 2-fold approach. At the same time, the exploration of different strategies to study a specific microbial population enhances the research process, providing in turn the opportunity to address new objectives in the *in vitro* and *in silico* representations of microbial systems and a more profound understanding of community dynamics.

## Author contributions

All authors were involved in the writing and proofing of the manuscript. RC, XP, and MG were responsible for the design, execution and interpretation of the experimental results. MB and MG were responsible for the statistical analysis of the experimental data. XP and MG were responsible for the modeling and simulation. All authors have read and approved the final manuscript.

### Conflict of interest statement

The authors declare that the research was conducted in the absence of any commercial or financial relationships that could be construed as a potential conflict of interest.
