# Supplementary material for: Digital Image Analysis of Yeast Single Cells Growing in Two Different Oxygen Concentrations to Analyze the Population Growth and to Assist Individual-Based Modeling
Source: Front Microbiol. 2018 Jan 4;8:2628. doi: 10.3389/fmicb.2017.02628 (PMC5758558; doi:10.3389/fmicb.2017.02628)
Supplement: Supplementary file 1 [file DataSheet1.PDF]

Supplementary Material

Digital image analysis of yeast single cells growing in two different oxygen concentrations to analyze the population growth and to assist individual-based modeling

Marta Ginovart[1,*], Rosa Carbó[2], Mónica Blanco[1], Xavier Portell[3]

1 Department of Mathematics, Universitat Politècnica de Catalunya, ESAB-Edifici D4, Esteve Terradas 8, 08860-Castelldefels, Barcelona, Spain
2 Department of Agri-Food Engineering and Biotechnology, Universitat Politècnica de Catalunya, ESAB-Edifici D4, Esteve Terradas 8, 08860-Castelldefels, Barcelona, Spain
3 Cranfield Soil and Agrifood Institute, Cranfield University, Bedfordshire MK43 0AL, United Kingdom

*** Correspondence:** Corresponding Author: marta.ginovart@upc.edu

S1. Sonication versus non-sonication experiment

Saccharomyces cerevisiae was grown in a flask of capacity 100 ml with 80 mL of glucose broth (Sharlab, Spain) for 40 hours at 27ºC. Once grown, and in aseptic conditions, a simple test was run to assess the robustness of the visual inspection of the digital image analysis described in the material and methods section for the identification of budded cells and unbudded cells. Two samples were taken from the growing culture. The first sample was diluted in a sterile saline solution and homogenized using a Vortex mixer (according to the material and methods section of this work), while the second sample (the rest of the suspension) was sonicated for 15 seconds, at 70W and 20 KHZ (Bandelin sonopuls GM70, Germany). A set of about 350 cells was inspected for each experimental condition in order to identify budded cells and unbudded cells. The classification process followed the same criteria explained in the material and methods section of the main manuscript. The cross tabulation of the counts of the four groups of data obtained (Sonication-Budded, Sonication-Unbudded, Non-sonication-Budded, Non-sonication-Unbudded) is shown in Table S1. These data were analyzed statistically by means of a Chi-square test for independence. This test was used to determine whether there was a significant association between the two variables. The statistical results suggested that the variables were not related at the 5% significance level (p-value = 0.144 according to the Pearson Chi-Square test, p-value = 0.162 according the Fisher’s exact test). Therefore, according to the test, the hypothesis of no association between the

two variables cannot be rejected at the 5% significance level.

Supplementary Table S1. Cross tabulation with the counts of the four groups Sonication-Budded cells, Sonication-Unbudded cells, Non-sonication-Budded cells, Non-sonication-Unbudded cells.

	Sonication	Non-sonication
Budded cells	83	92
Unbudded cells	283	243

S2. Experimental distributions of the following morphological variables of yeast cells: perimeter, major diameter, minor diameter, elongation (or aspect ratio) and circularity

The temporal distributions of the cell perimeters (Figure S2.1) display in general similar behaviors to those observed with the cell areas (Figure 5).

Figure S2.2 presents histograms of the major and minor diameter of the cells. Under aerobic conditions, the values of the minor diameters present unimodal distributions with high percentages for values between 5 and 7 µm, with only few changes depending on the growth phase. Nevertheless, there is a slight shift of higher percentages to high values during the log phase, and then a decline in the stationary phase. Under microaerophilic conditions, the minor diameters initially follow a unimodal distribution, and as the lag phase progresses, the distribution becomes rectangular with a range of percentages for values between 5 and 8.5 µm. In the log phase the distribution reverts to unimodal with higher percentages for minor diameters between 6 and 7.5 µm, and in the stationary phase the values of the minor diameters decrease (higher percentages correspond to diameters between 5 and 7 µm). The distributions of the major diameters vary in a more noticeable way depending on the growth phase. In aerobic conditions, they proceed from unimodal distributions in the samples corresponding to the lag phase (with sizes similar to the minor diameters, less than 8 µm) to rectangular distributions in the log phase samplings, in which a large part of the population increases the size of the major diameter and may reach up to 13 µm. Finally, in the samples corresponding to the stationary phase, bimodal distributions were observed, with the predominant peak displaced again to the left with diameters similar to the values of the minor diameters, and therefore reducing again its size. Under microaerophilic conditions, the

distributions of the major diameters vary more visibly depending on the growth phase, although to a lesser extent than in aerobic conditions.

The temporal evolutions of the variables estimating shape, namely, elongation (or aspect ratio) and circularity are displayed in Figures S2.3 and S2.4, respectively. It can be seen in Figure S2.3 that, at the beginning of the aerobic temporal evolution, in the lag phase, it is observed that the values of elongation fall below 1.5, whereas right at the beginning of the log phase this boundary is exceeded. The percentage of cells with values higher than 1.5 increases along the log phase and decreases again on reaching the stationary phase, yet without completely vanishing. The temporal distributions of the elongation of the cells growing under microaerophilic conditions are similar to bimodal distributions. In the lag phase there is a higher percentage of cells with elongations lower than 1.5, but during the log phase the higher percentages correspond to values greater than 1.5. At the end of the log phase and beginning of the stationary phase there is a tendency to reverse the percentages again, that is, a small decrease in the proportion of cells with high elongations can be observed. The temporal distributions of the parameter circularity in aerobic conditions evolve according to the population growth phase (Figure S2.4). In the lag phase, the values range from 0.6 to 0.9, with a higher percentage of cells concentrated between 0.8 and 0.9. As the log phase progresses the percentages are reversed so that cells with values around 0.7 dominate. In the stationary phase, the percentages are inverted again, with values close to 1. In microaerophilic conditions, the lag phase is also characterized by a circularity range between 0.6 and 0.9. But unlike in aerobic growth, in this case no percentage for particular values seems remarkable stand out. During the log phase the percentage of cells farthest from the circular shape increases and in the stationary phase, the results tend to be similar to the ones obtained in the lag phase.

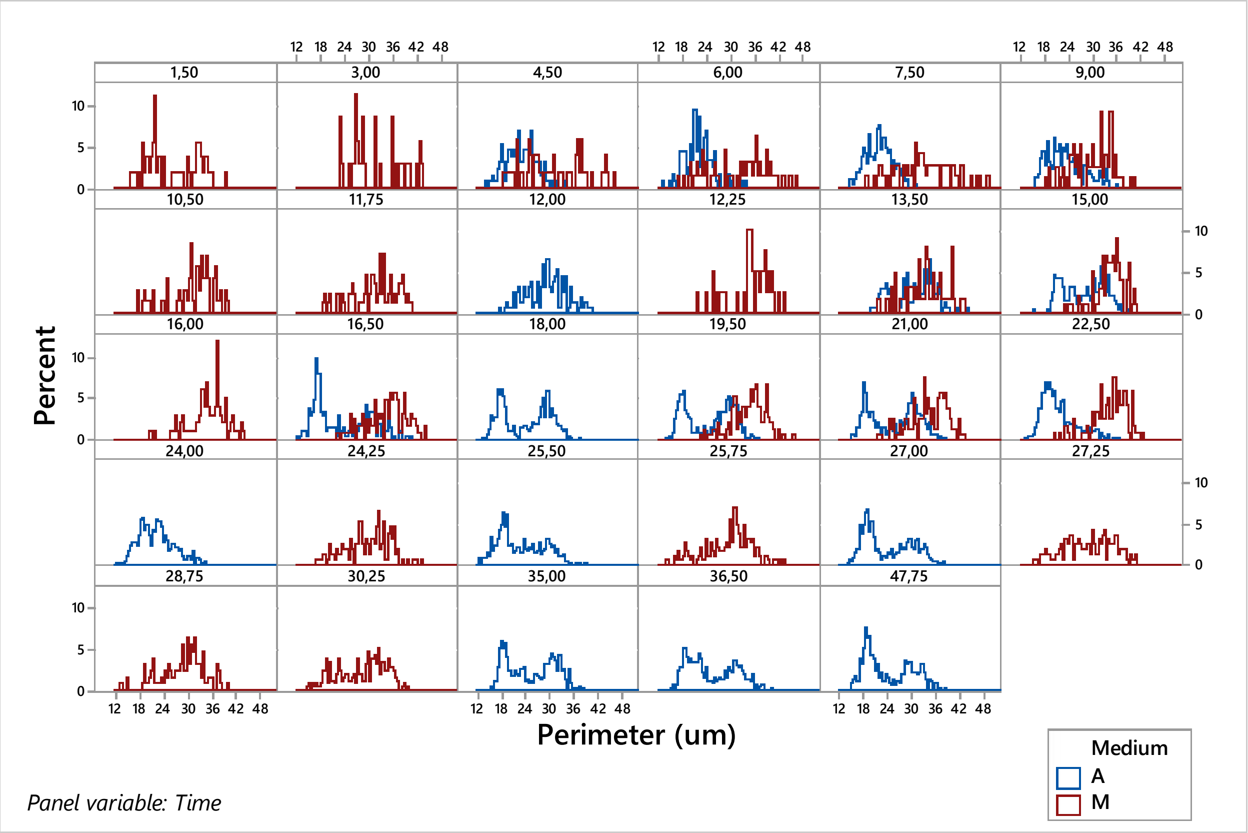

Supplementary Figure S2.1. Histograms of the perimeter variable for the pooled data of the four replicates under aerobic conditions (A) and of the four replicates under microaerophilic conditions (M) corresponding to different sampling times.

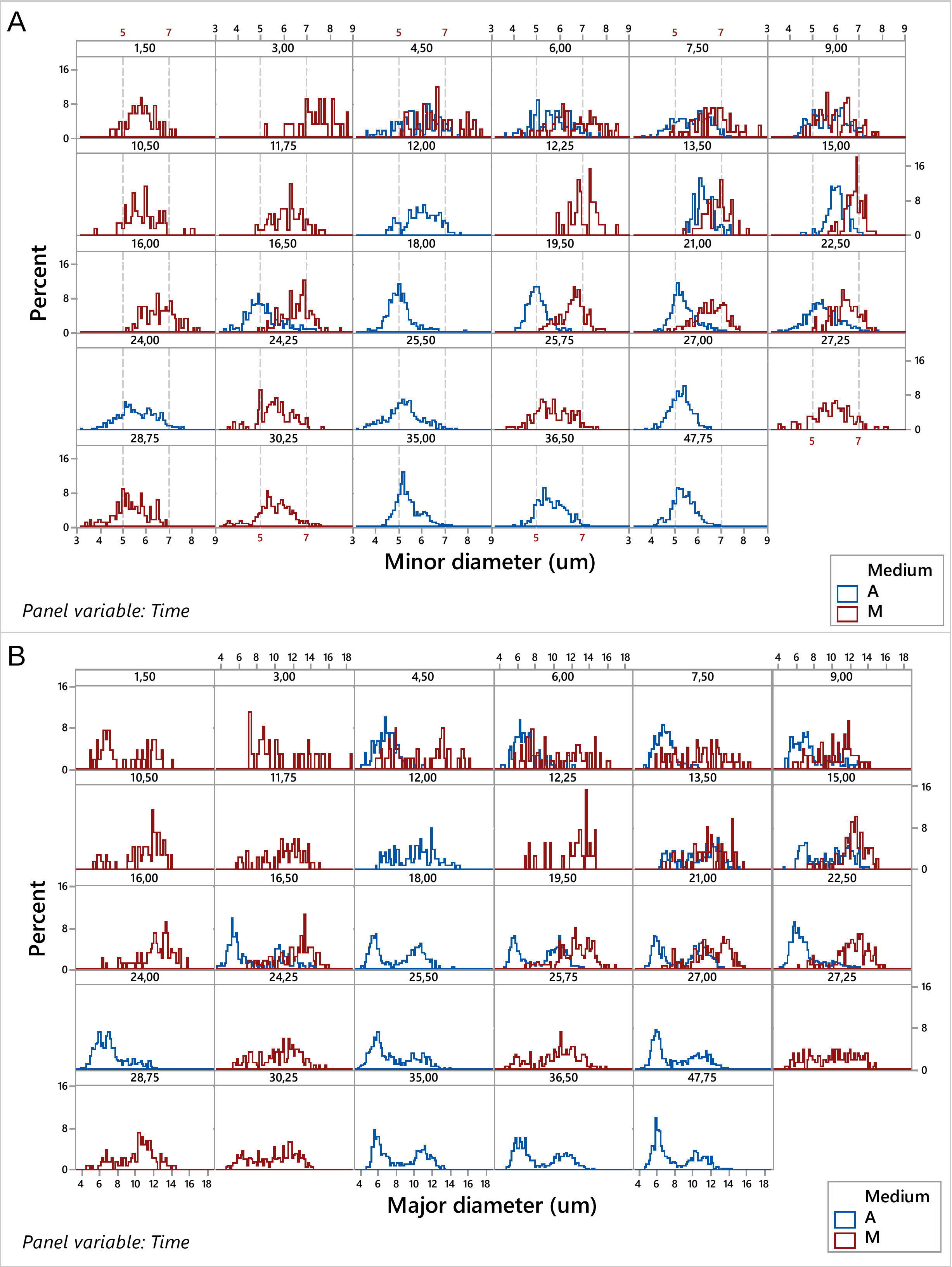

Supplementary Figure S2.2. Histograms of the variables minor diameter (A-top) and major diameter (B-bottom) for the combined data of the four replicates under aerobic conditions and of the four replicates under microaerophilic conditions corresponding to different sampling times.

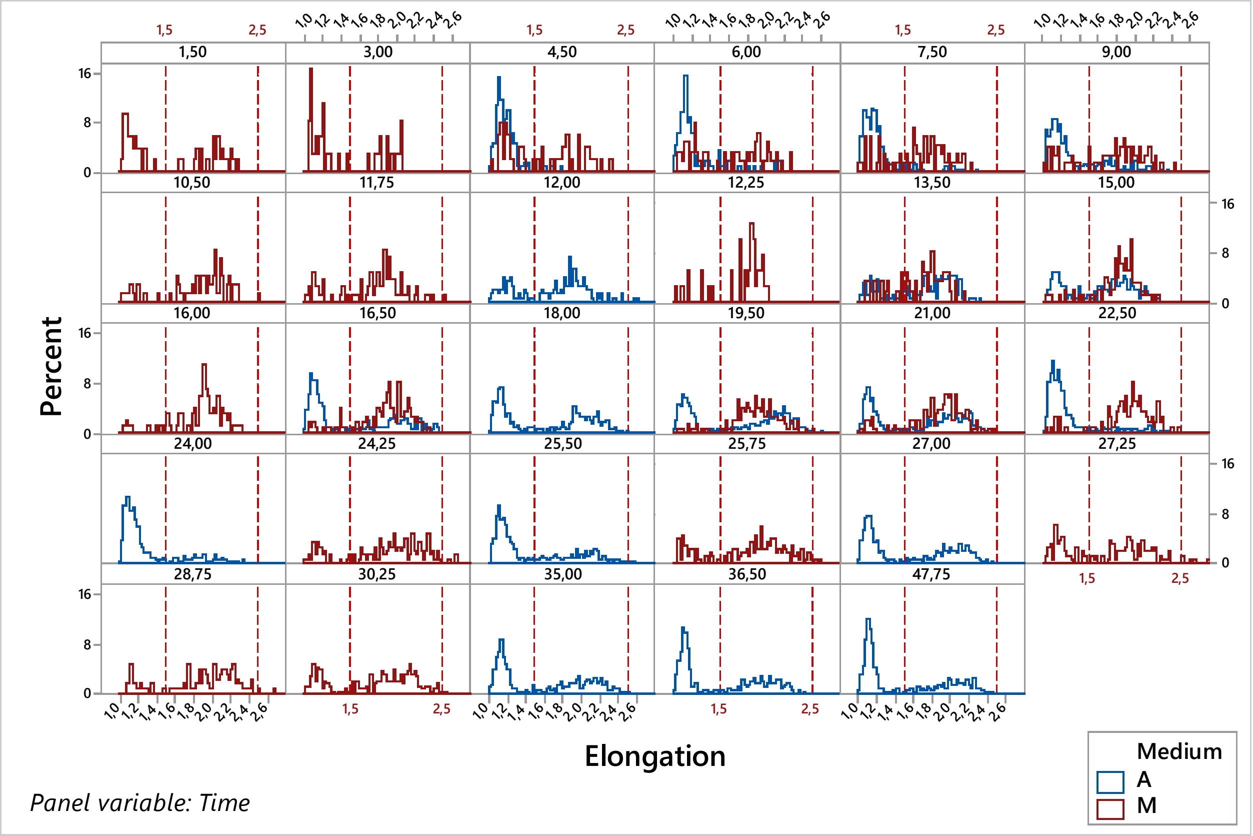

Supplementary Figure S2.3. Histograms of the elongation (or aspect ratio) variable for the pooled data of the four replicates under aerobic conditions (A) and of the four replicates under microaerophilic conditions (M) corresponding to different sampling times.

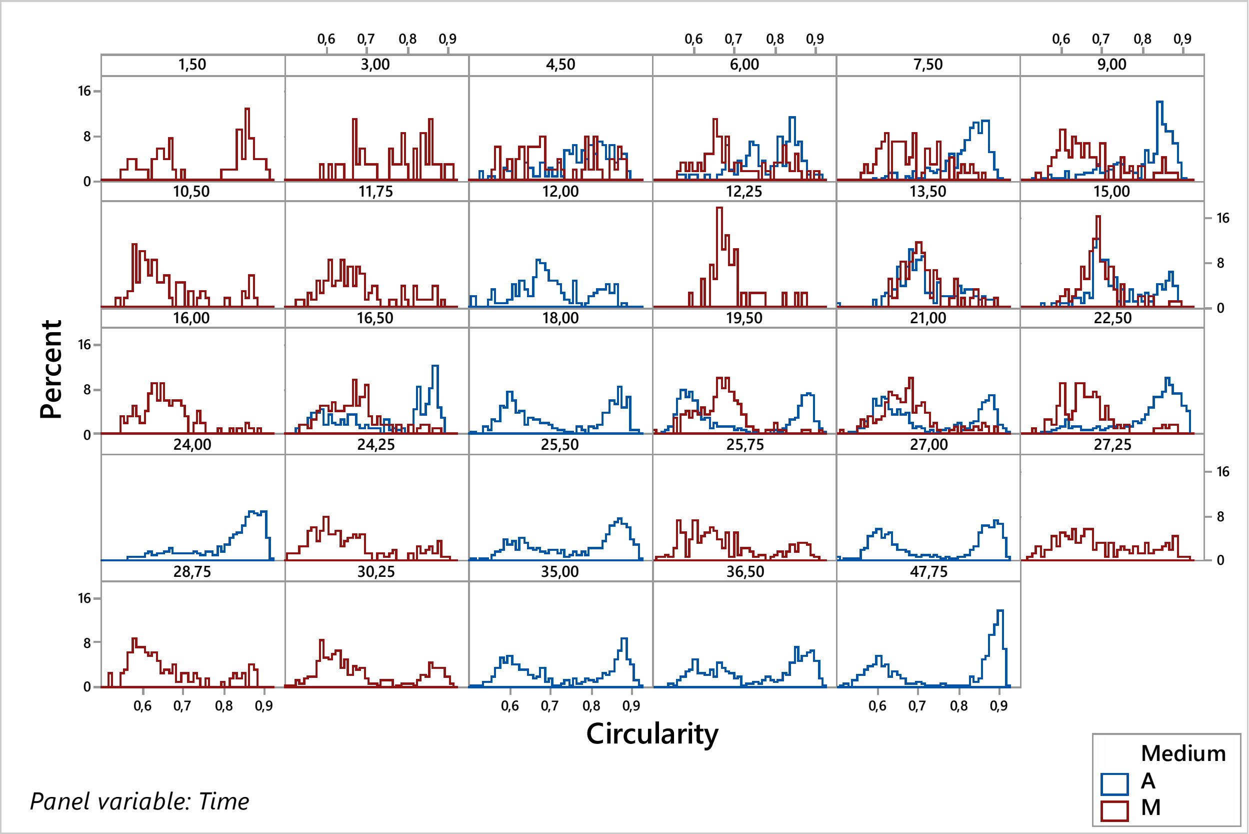

Supplementary Figure S2.4. Histograms of the circularity variable for the pooled data of the four replicates under aerobic conditions (A) and of the four replicates under microaerophilic conditions (M) corresponding to different sampling times.
